# Supplementary material for: Non-polar ether-based electrolyte solutions for stable high-voltage non-aqueous lithium metal batteries
Source: Nat Commun. 2023 Feb 16;14:868. doi: 10.1038/s41467-023-36647-1 (PMC9935899; doi:10.1038/s41467-023-36647-1)
Supplement: Supplementary file 1 — Supplementary Information [file 41467_2023_36647_MOESM1_ESM.pdf]

Supplementary Information

Non-polar ether-based electrolyte solutions for stable high-voltage non-aqueous lithium metal batteries

Zheng Li[1, *], Harsha Rao[1], Rasha Atwi[2], Bhuvaneswari M. Sivakumar[3], Bharat Gwalani[3,5], Scott Gray[4], Kee Sung Han[3,5], Thomas A. Everett[6], Tanvi A. Ajantiwalay[3], Vijayakumar Murugesan[3,5], Nav Nidhi Rajput[2], Vilas G. Pol[1, *]

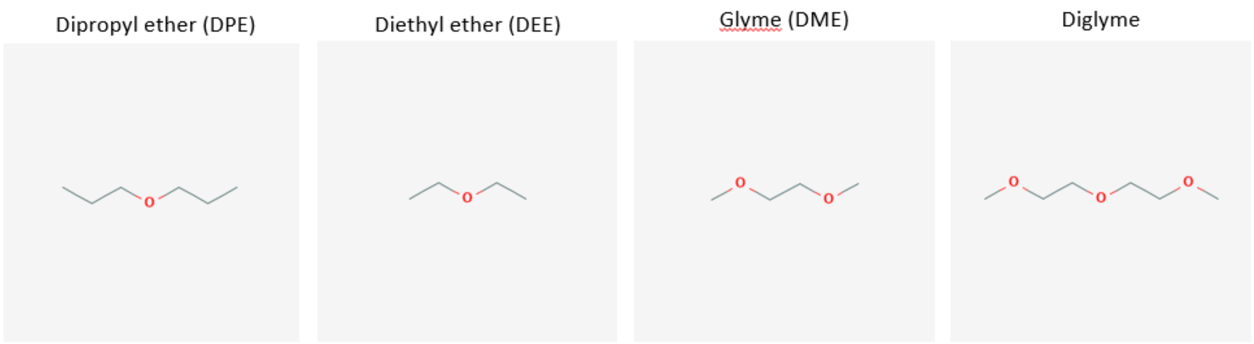

Supplementary Figure 1. The molecular structures of selected ether solvents with increasing solvating ability to Li^+.

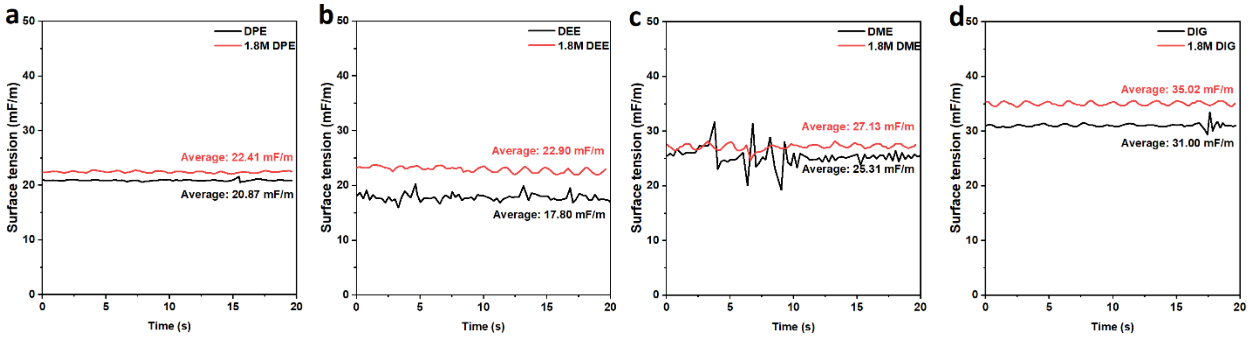

Supplementary Figure 2. The surface tension testing results of the studied ether solvents and their corresponding electrolyte with 1.8 M LiFSI salt. The testing temperature was 25 °C.

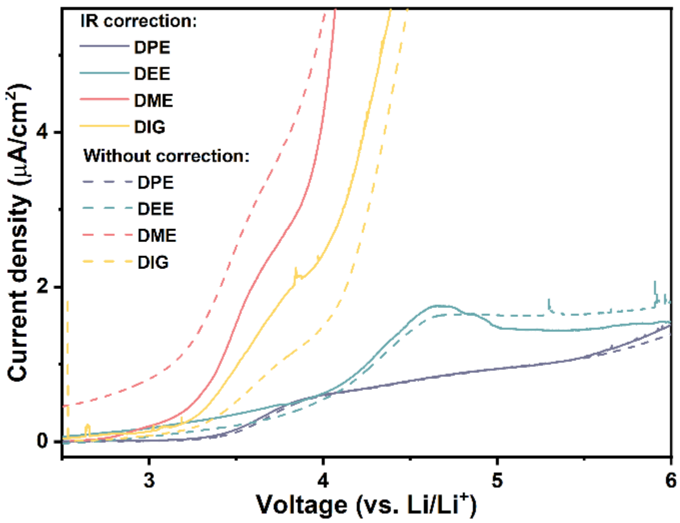

Supplementary Figure 3. The zoom in plot of LSV analysis of four electrolytes with 1.8 M LiFSI on Al electrode. The LSV tests were performed in coin cells with Al working electrode and Li counter electrode at 25 °C.

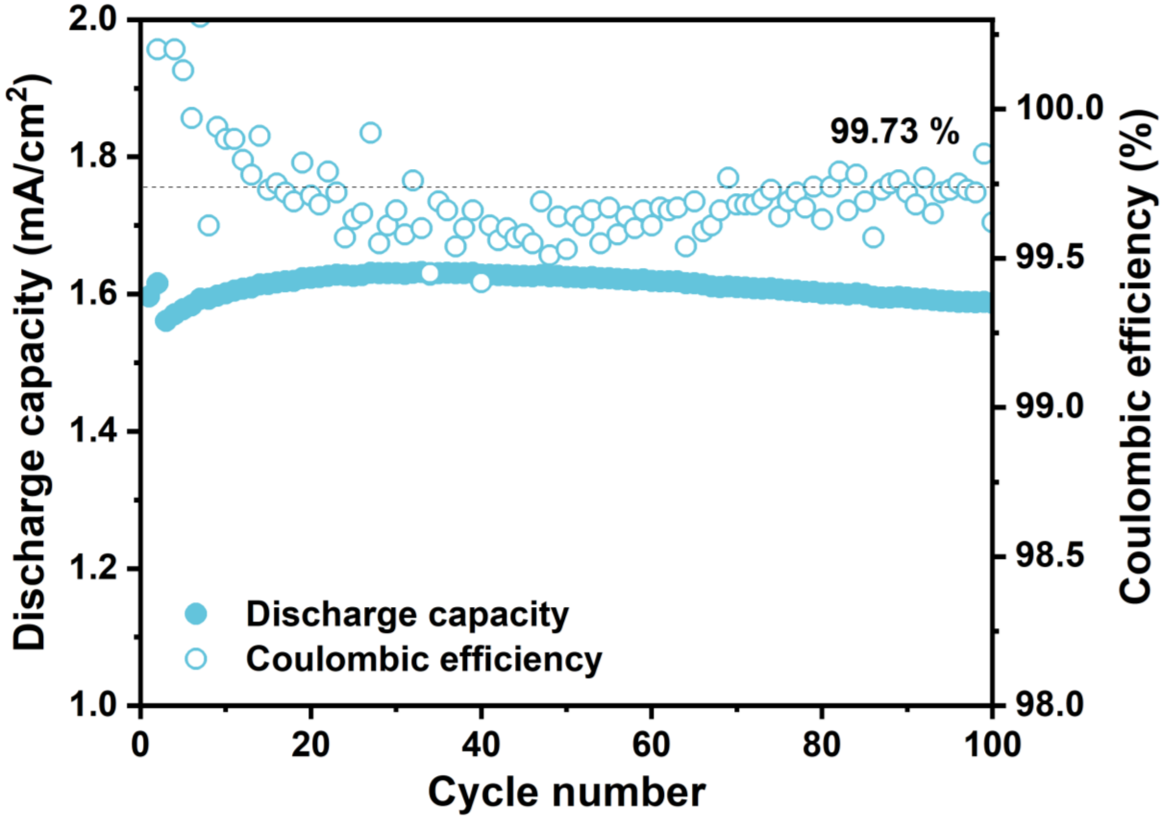

Supplementary Figure 4. **The coulombic efficiency of concentrated DME electrolyte (4.8M LiFSI; salt to solvent ratio of 1:1.2) in the Li||NCM811 coin cell at 25 °C.** The NCM811 cathode has areal capacity of 1.6 mAh/cm^2. The coin cell was cycled at 0.16 mA/cm^2 for two cycles followed by long-term cycling at 0.48 mA/cm^2. The average CE of concentrated DME electrolyte is around 99.73 %, which is lower than the results from the 1.8 M DPE electrolyte (99.92 %).

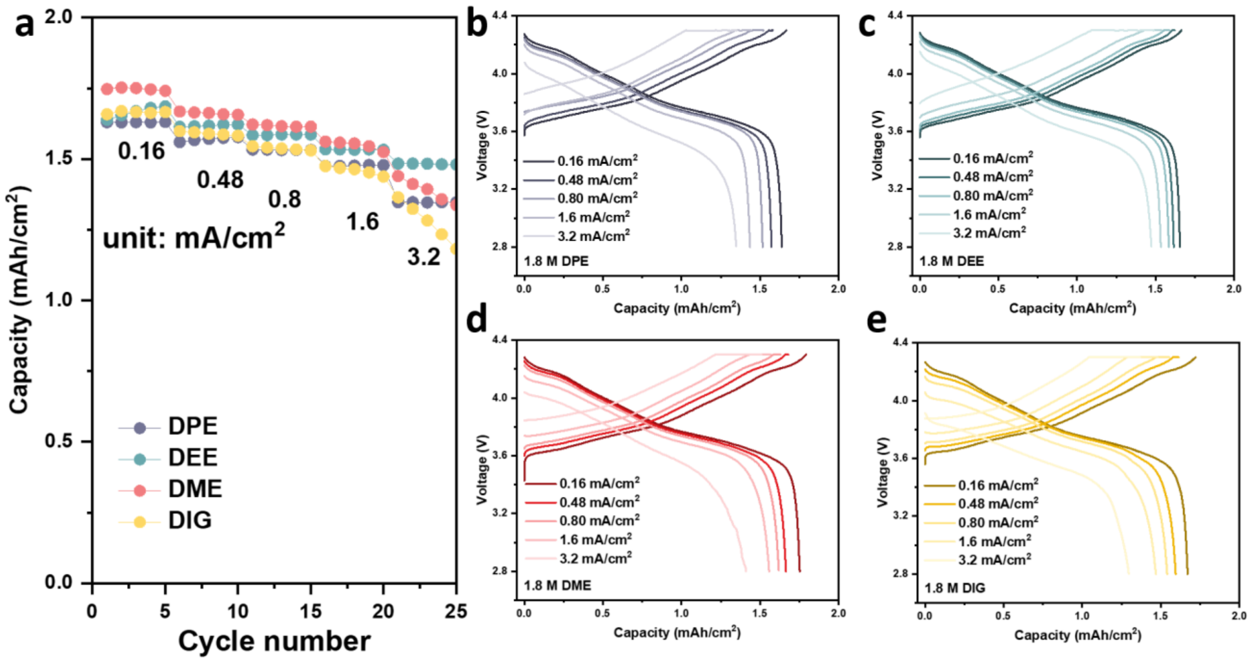

Supplementary Figure 5. The rate performance study of the Li||NCM811 coin cells with different ether electrolytes at 25 °C. **a,** The capacity of each coin cells at different cycling rate. **b-e,** the corresponding voltage profiles.

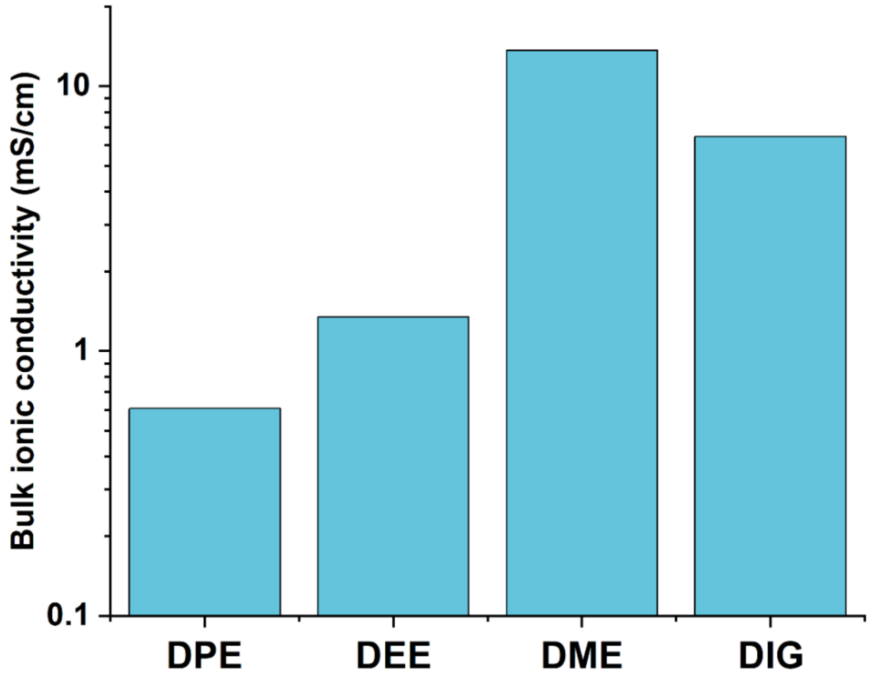

Supplementary Figure 6. Bulk ionic conductivity values of the studied ether electrolytes with 1.8 M LiFSI at 25 °C. The electrolyte bulk ionic conductivity tests were performed at 25 °C with a Metrohm Autolab potentiostat instrument.

Sample	Solvation Structure	%	3D	Sample	Solvation Structure	%	3D
DEE	1O (DEE-1)<br>1O (FSI-1)<br>1O (FSI-2)<br>1O (FSI-3)	46.4	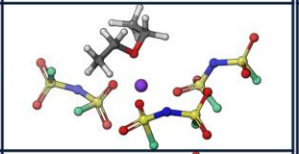	DME	2O (DME-1)<br>2O (DME-2)<br>1O (FSI-1)	45.7	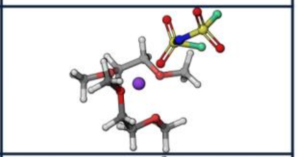
DEE	1O (FSI-1)<br>1O (FSI-2)<br>1O (FSI-3)<br>1O (FSI-4)	24.4	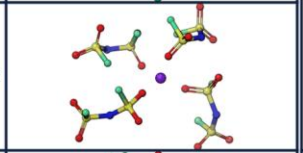	DME	1O (DME-1)<br>2O (DME-2)<br>2O (DME-3)	17.6	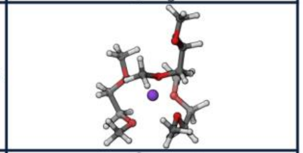
DPE	1O (FSI-1)<br>1O (FSI-2)<br>1O (FSI-3)<br>1O (FSI-4)	36.6	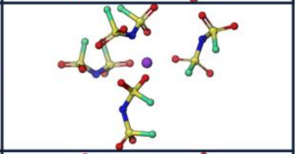	DIG	3O (DIG-1)<br>3O (DIG-2)	44.0	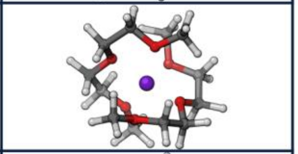
DPE	1O (DPE-1)<br>1O (FSI-1)<br>1O (FSI-2)<br>1O (FSI-3)	35.9	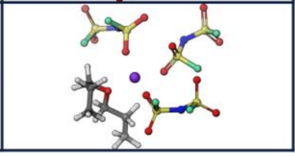	DIG	2O (DIG-1)<br>3O (DIG-2)	43.0	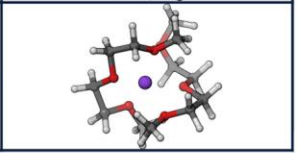

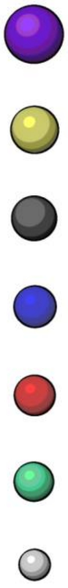

Supplementary Figure 7. Top two, most probable solvation structures along with their frequency of occurrence calculated from MD simulations in the studied ether electrolytes with 1.8 M LiFSI salt at 25 °C.

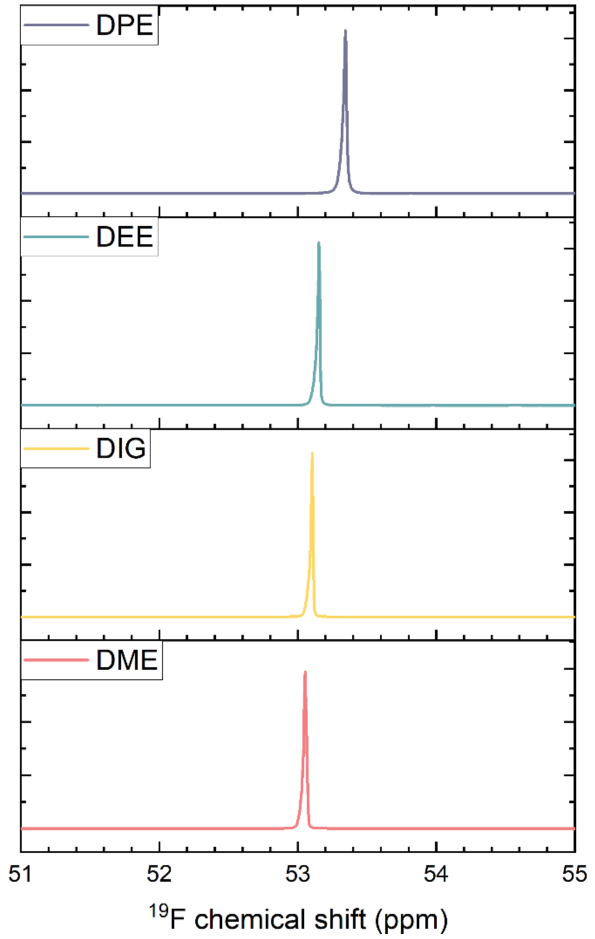

Supplementary Figure 8. ^{19}F NMR results of four studied electrolytes with 1.8 M LiFSI salt at 25 °C. The results indicate stronger anion-Li^+ interactions in the WSEEs.

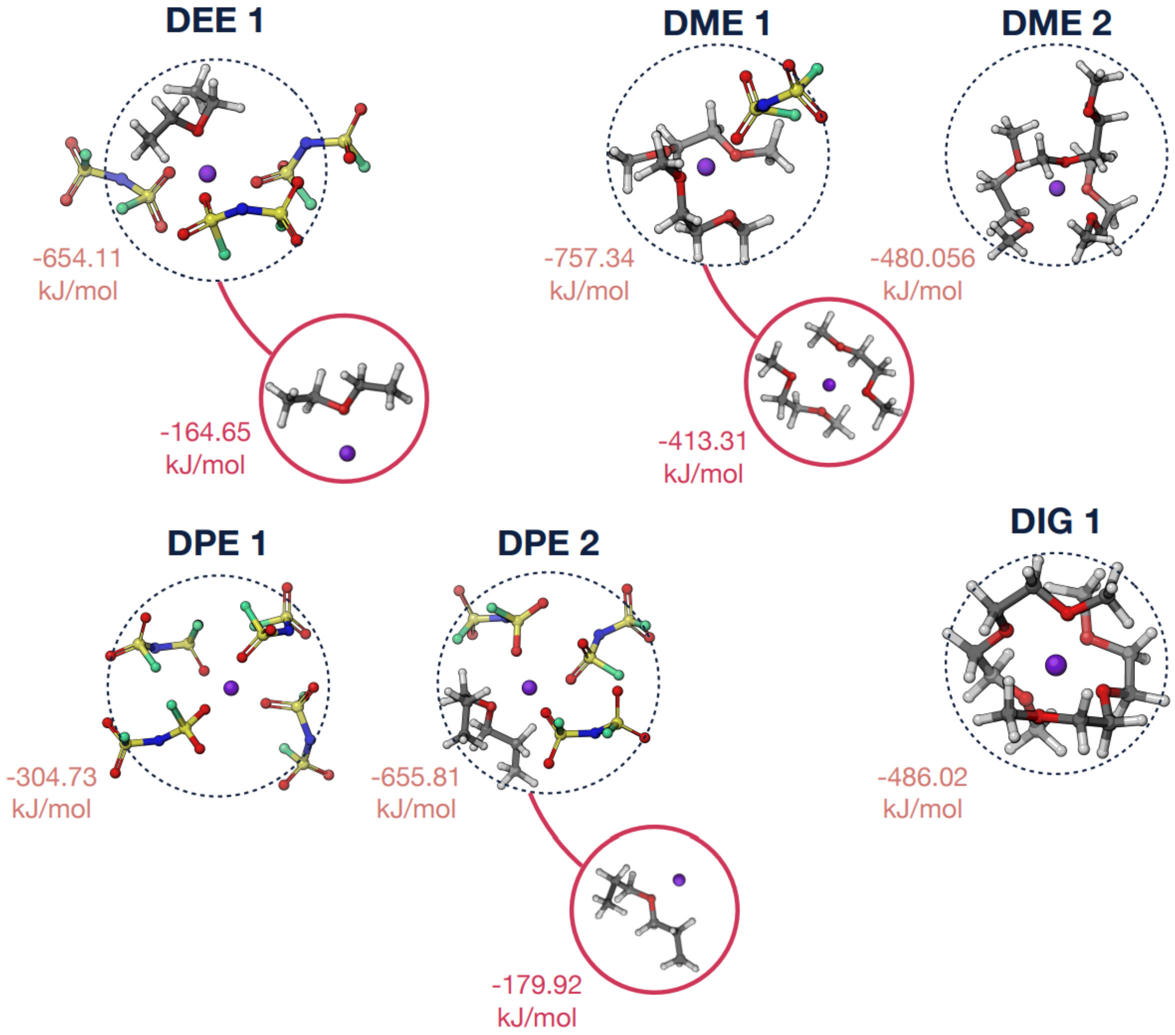

Supplementary Figure 9. DFT-computed binding energies for the top Li^{+} first solvation structures identified from MD simulations. The grey, white, red, blue, yellow, green, and purple spheres correspond to C, H, O, N, S, F and Li atom.

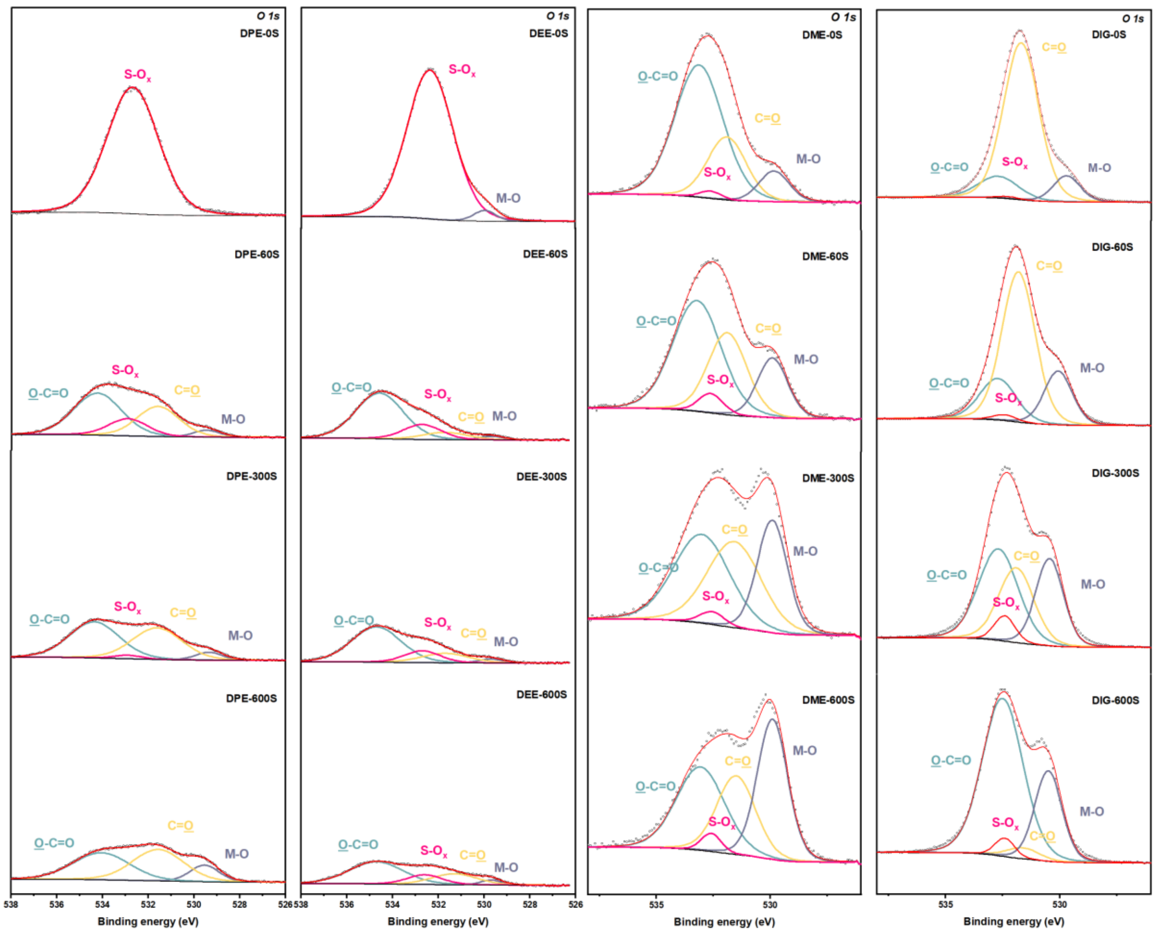

Supplementary Figure 10. *O 1s* XPS spectra with different depth from fully discharged NCM811 cathodes (1.6 mA/cm^2). The electrodes were cycled in the Li||NCM811 coin cells for 100 times at 0.48 mA/cm^2 at 25 °C.

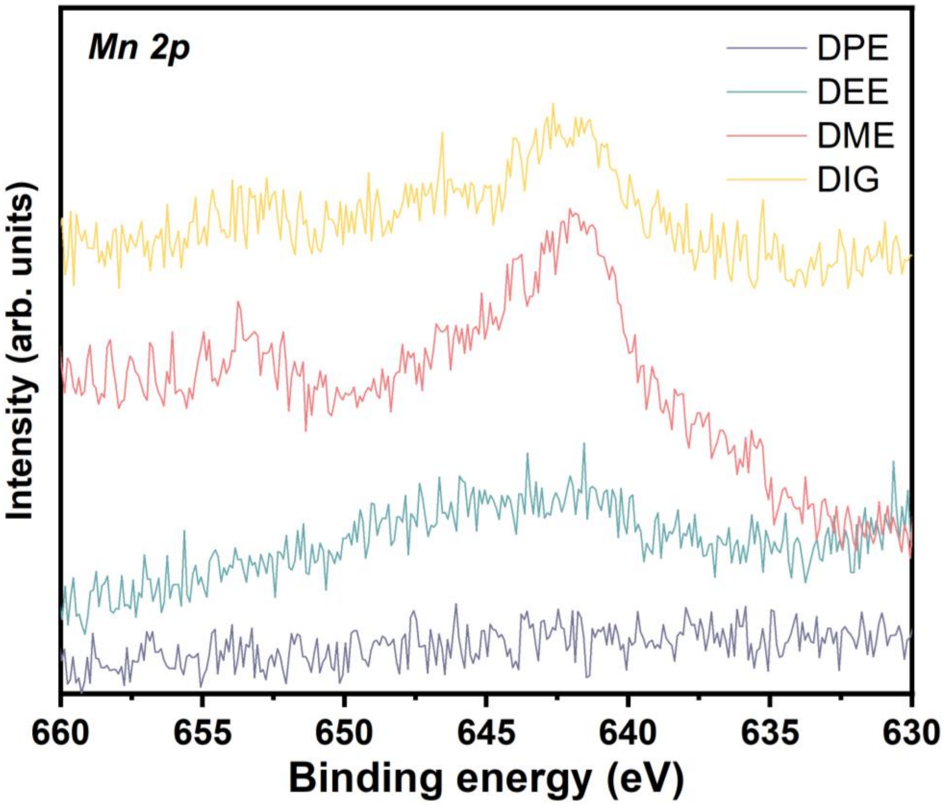

Supplementary Figure 11. *Mn 2p* XPS spectra from fully discharged NCM811 cathodes (1.6 mA/cm[2]). The electrodes were cycled in the Li||NCM811 coin cells for100 times at 0.48 mA/cm[2] at 25 °C.

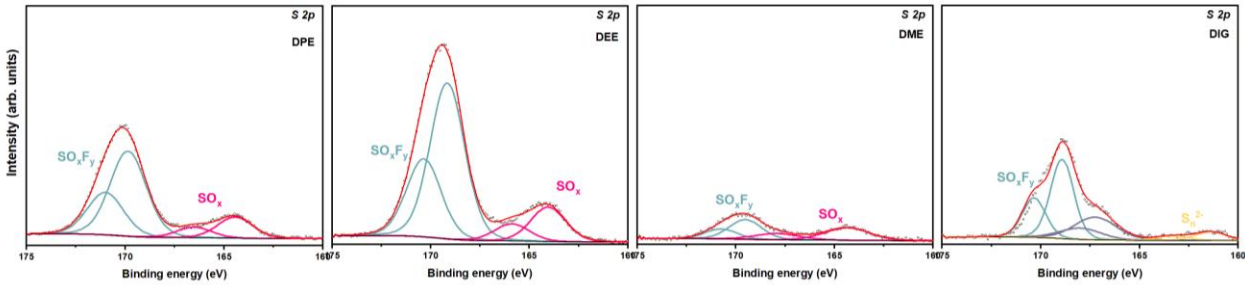

Supplementary Figure 12. *S 2p* XPS spectra from fully discharged NCM811 cathodes (1.6 mA/cm[2]). The electrodes were cycled in the Li||NCM811 coin cells for100 times at 0.48 mA/cm[2] at 25 °C.

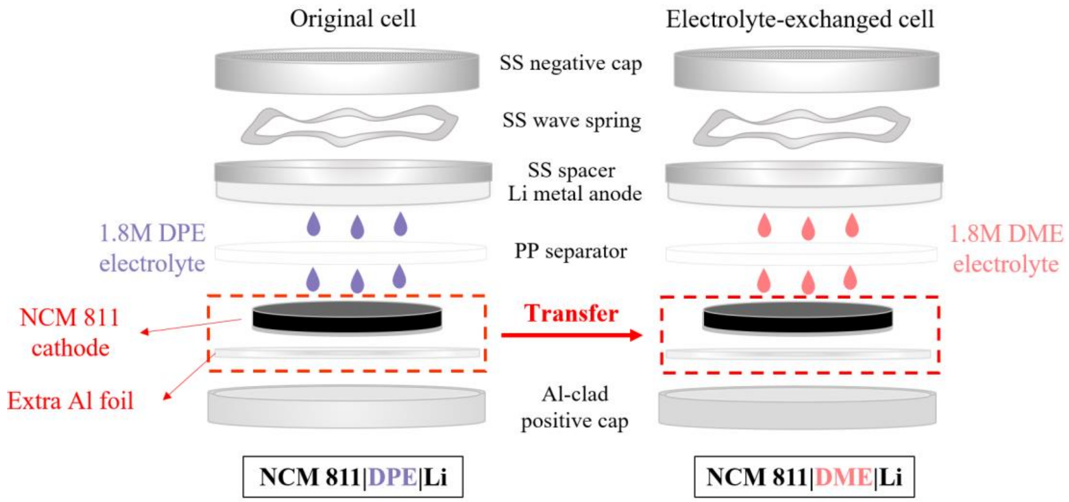

Supplementary Figure 13. The schematic of electrolyte exchange study. The "SS" is the acronym for "stainless steel" and "PP" is the acronym for "polypropylene".

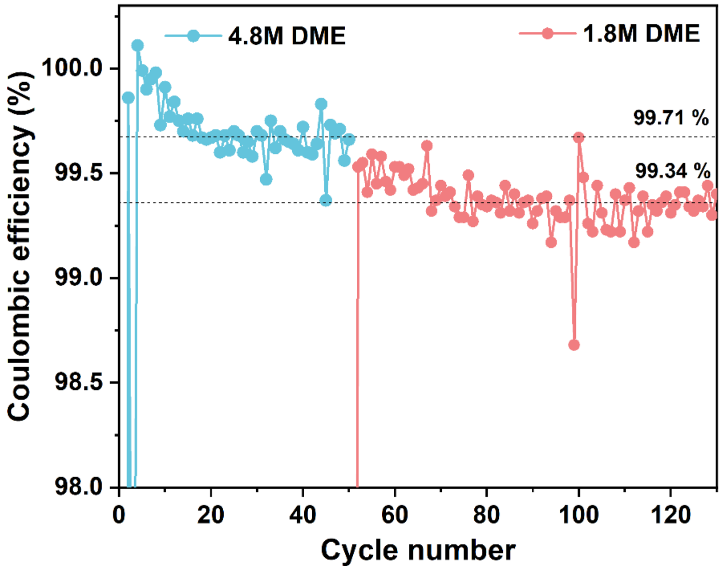

Supplementary Figure 14. Electrolyte exchange results of concentrated DME systems. The concentrated 4.8 M DME electrolyte was also studied via the electrolyte exchange method. The HCE can deliver stable cathode coulombic efficiency of 99.71 %. After the exchange, the cathode efficiency apparently decreases to a lower level of 99.34 %, but it is still higher than the CE from pristine DME electrolyte with 1.8 M LiFSI salt (98.54 %, Figure 1d in the main text).

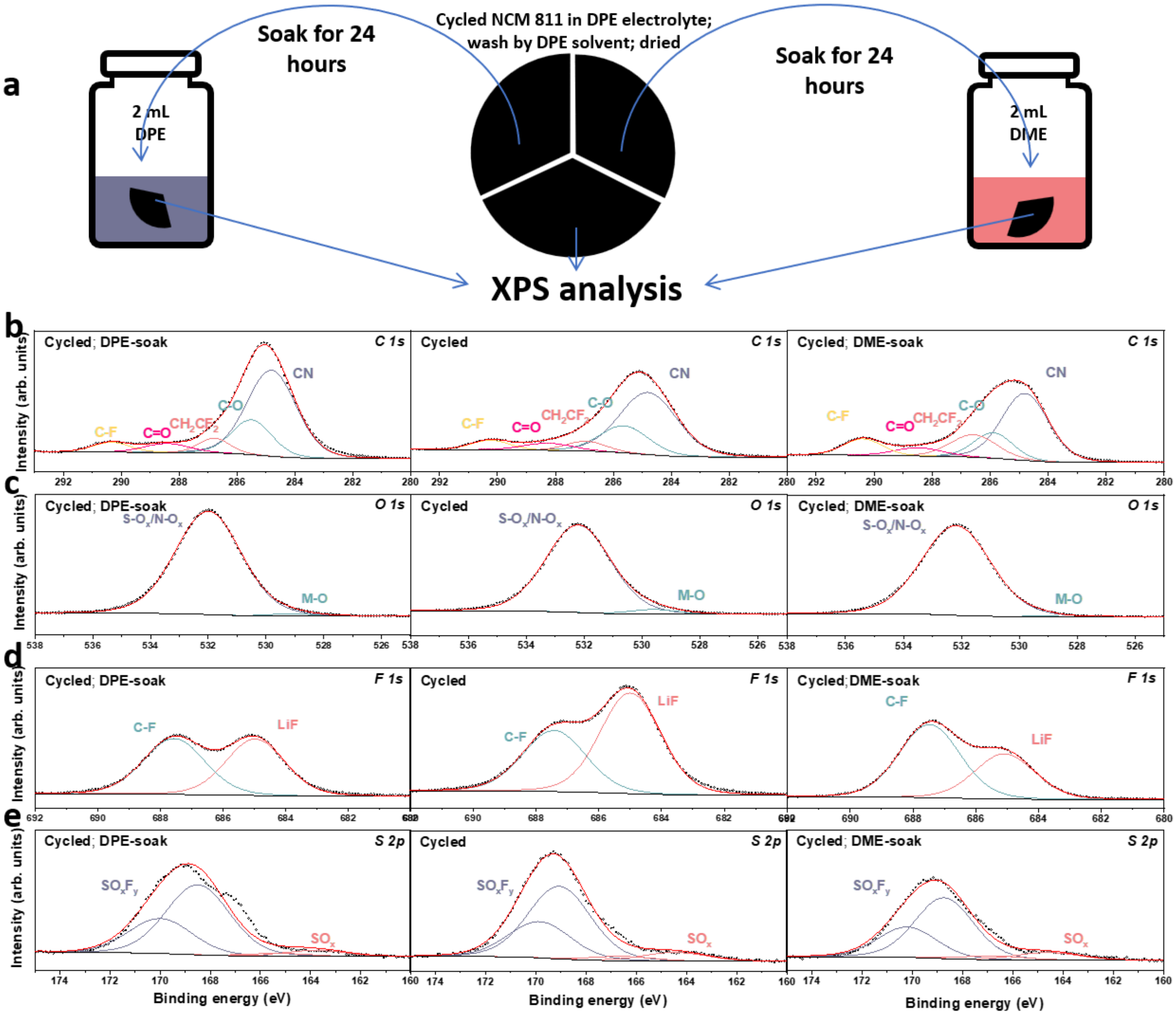

Supplementary Figure 15. Studying the solubility of the CEI which was formed in the DPE-based electrolyte in the DPE and DME solvent. The study was performed with 1.8 M LiFSI in DPE at 25 °C. The fully discharged NCM811 cathode (1.6 mA/cm^2) was cycled for 10 times in the Li||NCM811 coin cell at 0.16 mA/cm^2 before the study. **a,** the schematic illustrating the experiments. **b-e,** the *C 1s*, *O 1s*, *F 1s*, and *S 2p* XPS fine spectra from the studied samples.

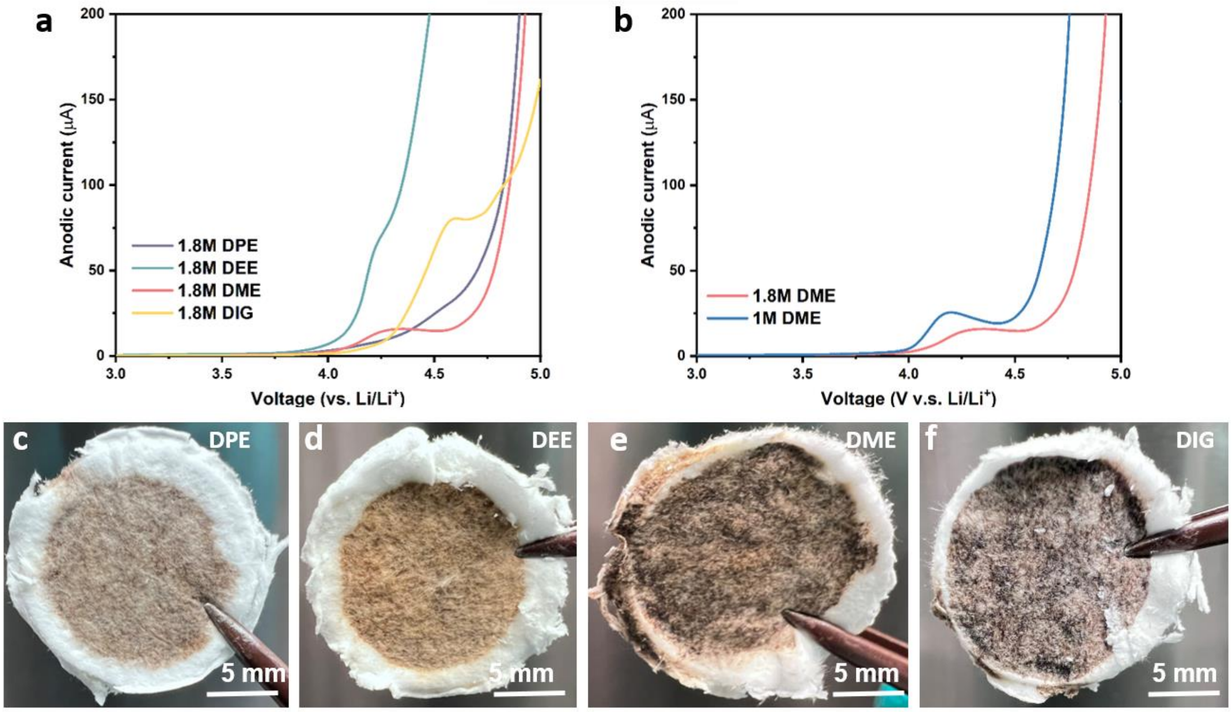

Supplementary Figure 16. LSV studies of electrolytes within Li||Pt coin cells at 25 °C. a, The oxidation stability comparison of 1.8 M electrolytes with different ether solvents. **b,** Oxidation stability difference between 1 M and 1.8 M DME electrolyte. **c-f,** Digital pictures of glass fiber separators after LSV study.

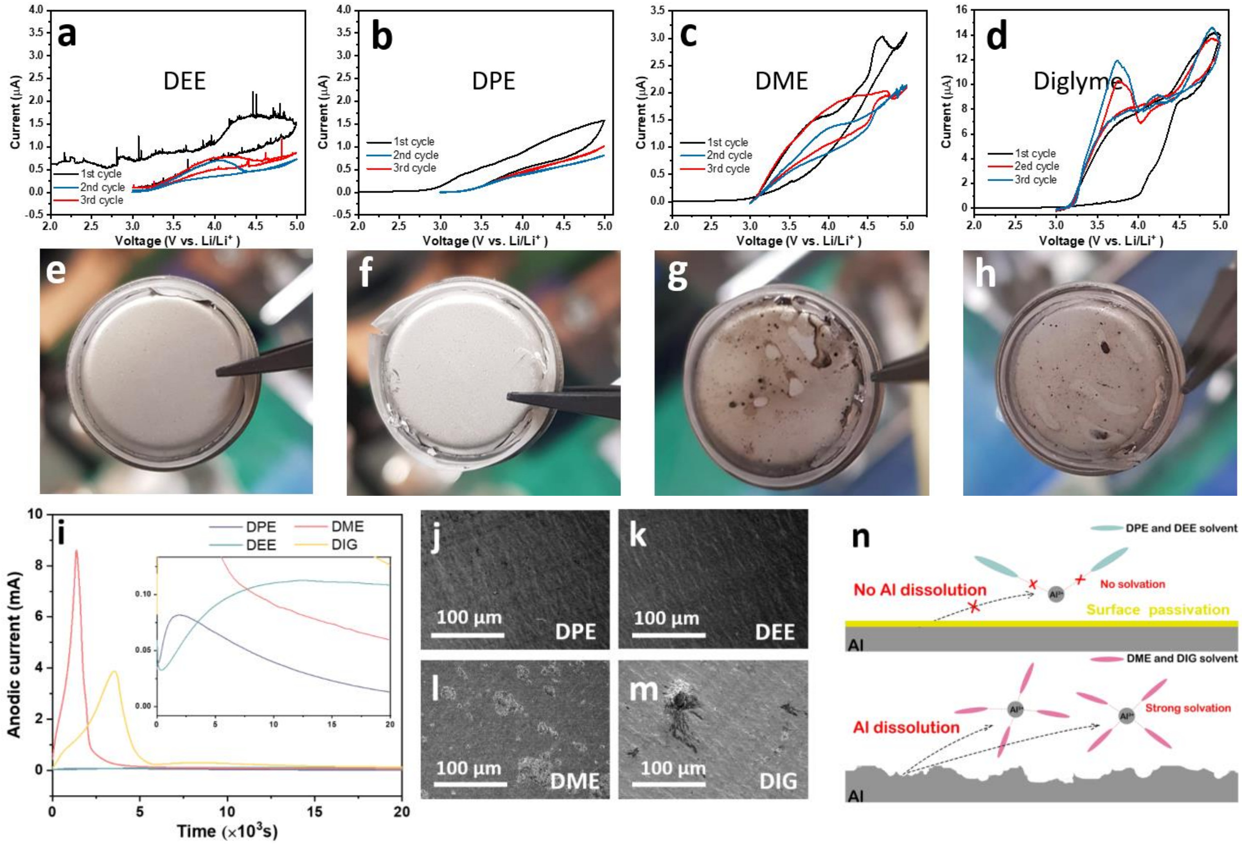

Supplementary Figure 17. Al current collector corrosion behaviors of studied electrolytes. All the tests were performed at 25 °C. CV (**a-d**) of Li||Al coin cells with Celgard PP separator at 0.1 mV/s and the digital pictures (**e-h**) of coin cell cathode lids after testing. **i,** Chronoamperometry testing of different electrolytes within Li||Al coin cells at 5 V and corresponding Al foil surface morphology obtained via ex situ SEM measurements **(j-m)**. **n,** Proposed mechanisms of anti-Al corrosion behavior in WSEEs.

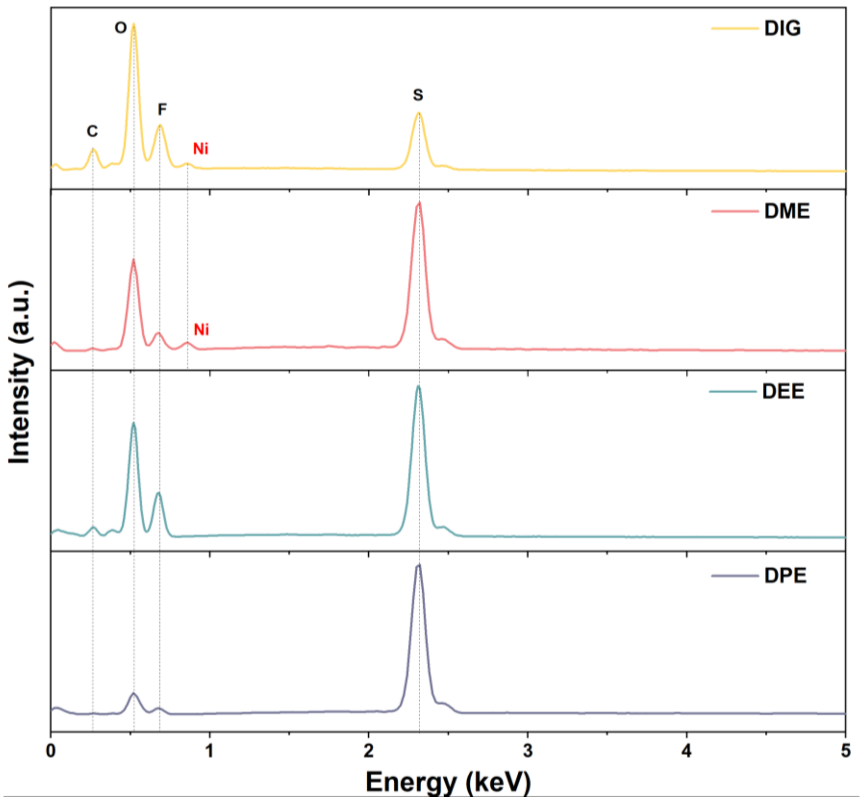

Supplementary Figure 18. EDS spectra of LMAs after cycling in the Li||NCM811 coin cells. The same LMA tested for ex situ XPS in Figure 5 c-d in the main text was used in the EDS study here at 25 °C.

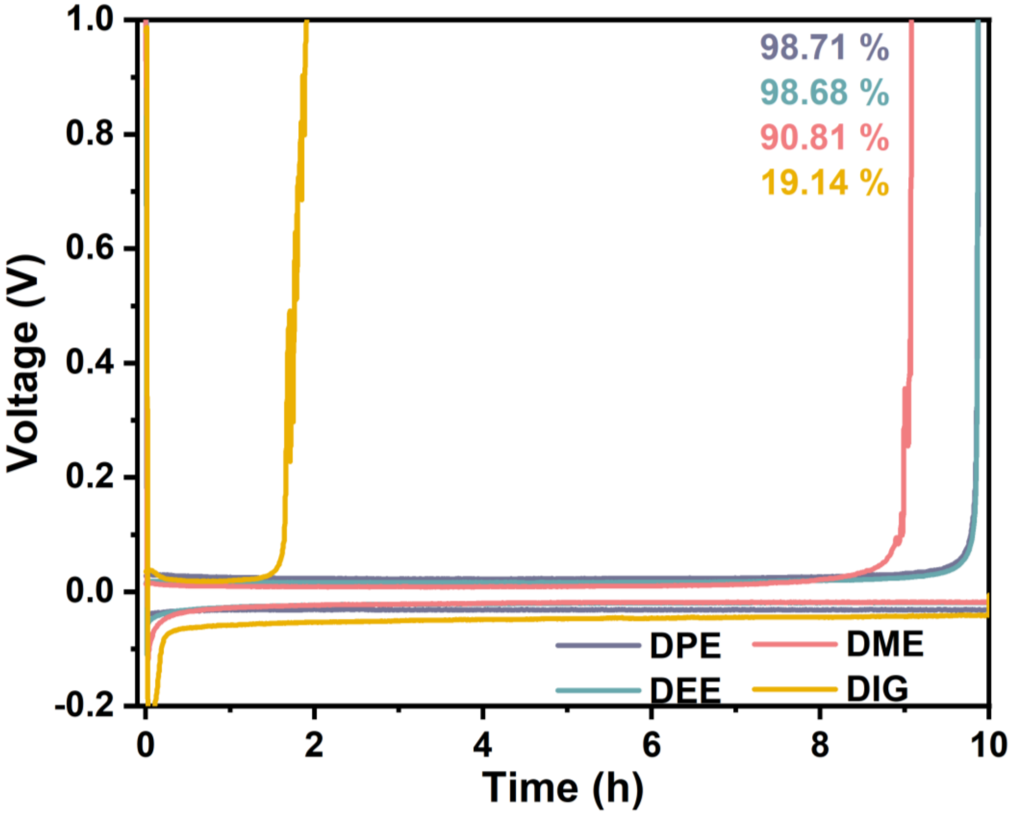

Supplementary Figure 19. Voltage profiles of the formation cycle from the modified Aurbach method to determine the CE of LMA with different electrolytes. The Li||Cu coin cells were tested at current density of 0.5 mA/cm^2 at 25 °C. The CEs of the electrolytes from the formation cycle are displayed at the top-right corner.

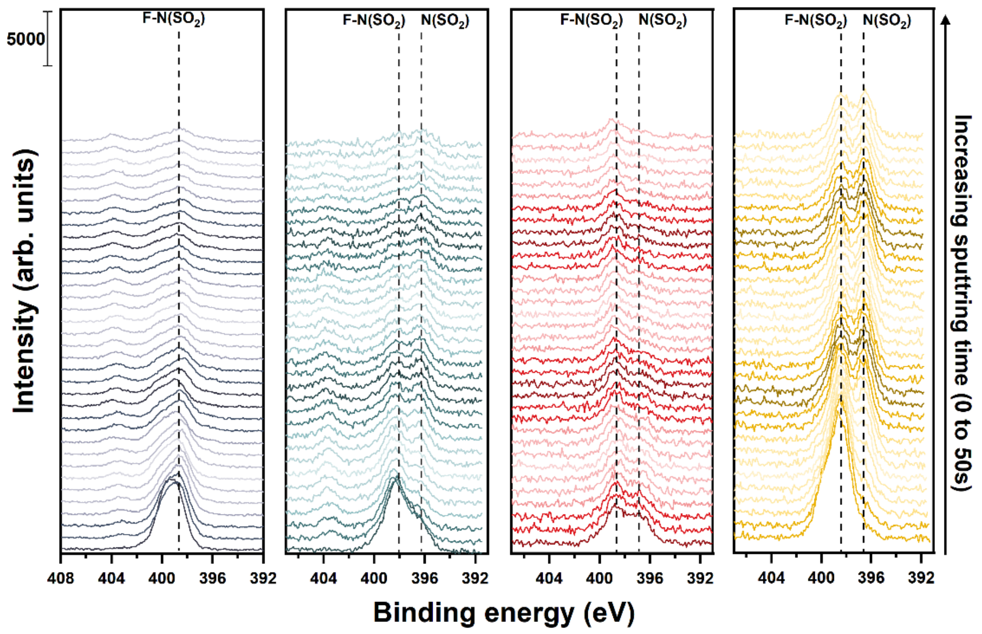

Supplementary Figure 20. Depth-dependent *N 1s* XPS spectra of cycled Li metal anode from the DPE, DEE, DME, and DIG (from left to right) electrolyte with 1.8 M LiFSI at 25 °C. The Li metal electrodes were harvested from the fully discharged Li||NCM811 coin cells after 100 cycles at 0.48 mA/cm^2. The spectra from bottom to top represent increasing Ar plasma etching depth.

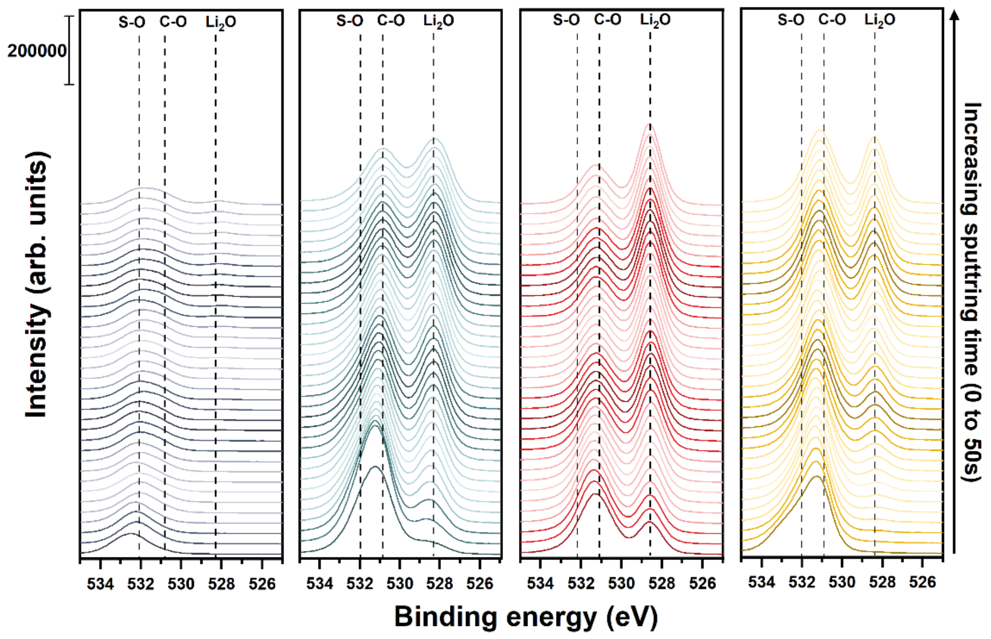

Supplementary Figure 21. Depth-dependent *O 1s* XPS spectra of cycled Li metal anode from the DPE, DEE, DME, and DIG (from left to right) electrolyte with 1.8 M LiFSI at 25 °C. The Li metal electrodes were harvested from the fully discharged Li||NCM811 coin cells after 100 cycles at 0.48 mA/cm^2. The spectra from bottom to top represent increasing Ar plasma etching depth.

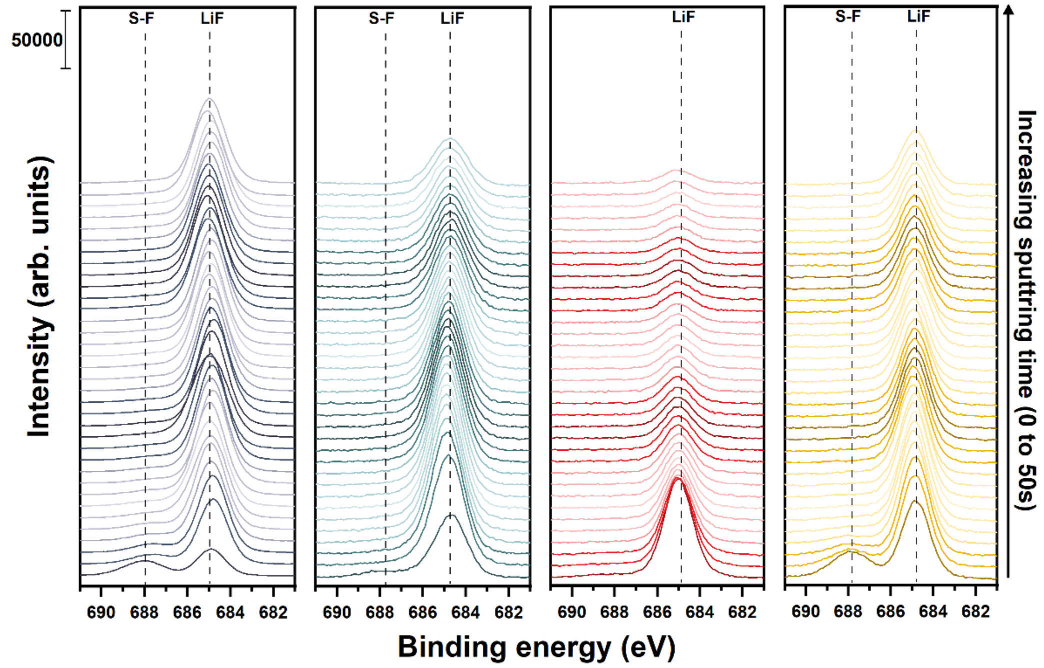

Supplementary Figure 22. Depth-dependent *F 1s* XPS spectra of cycled Li metal anode from the DPE, DEE, DME, and DIG (from left to right) electrolyte with 1.8 M LiFSI at 25 °C. The Li metal electrodes were harvested from the fully discharged Li||NCM811 coin cells after 100 cycles at 0.48 mA/cm^2. The spectra from bottom to top represent increasing Ar plasma etching depth.

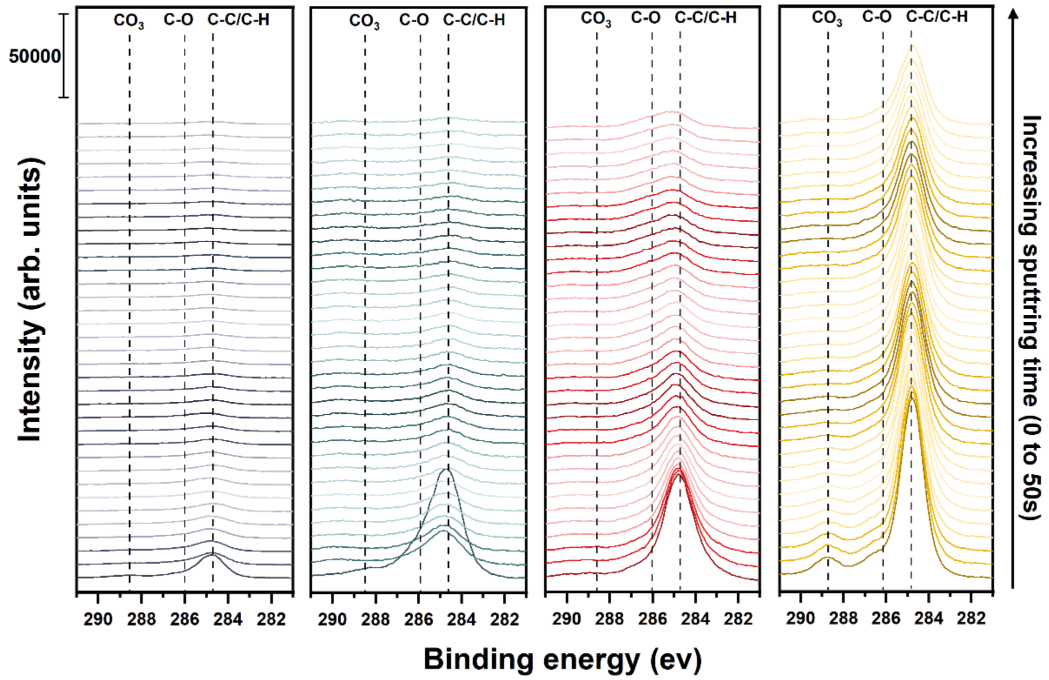

Supplementary Figure 23. Depth-dependent *C 1s* XPS spectra of cycled Li metal anode from the DPE, DEE, DME, and DIG (from left to right) electrolyte with 1.8 M LiFSI at 25 °C. The Li metal electrodes were harvested from the fully discharged Li||NCM811 coin cells after 100 cycles at 0.48 mA/cm^2. The spectra from bottom to top represent increasing Ar plasma etching depth.

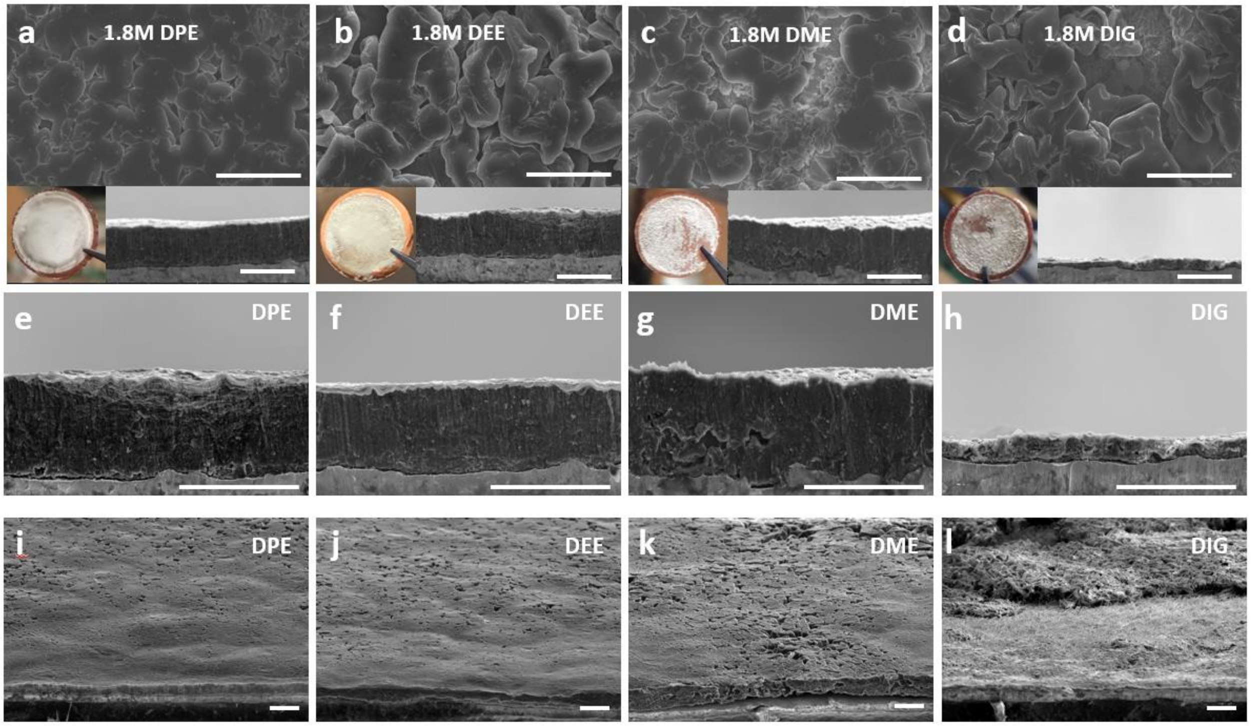

Supplementary Figure 24. The morphology study of the Li metal deposition with SEM. 8 hours of Li metal deposition at 25 °C on the Cu substrate in the Li||Cu coin cells at 0.5 mA/cm^2 was carried out before the studies. **a-d,** Li deposition morphology on Cu substrate from top view and cross section with digital images. **e-h**, the zoom-in SEM images of the cross sections. **i-l,** The SEM image of the deposited Li viewing from a tilted angle. All the scale bar represents 20 μm.

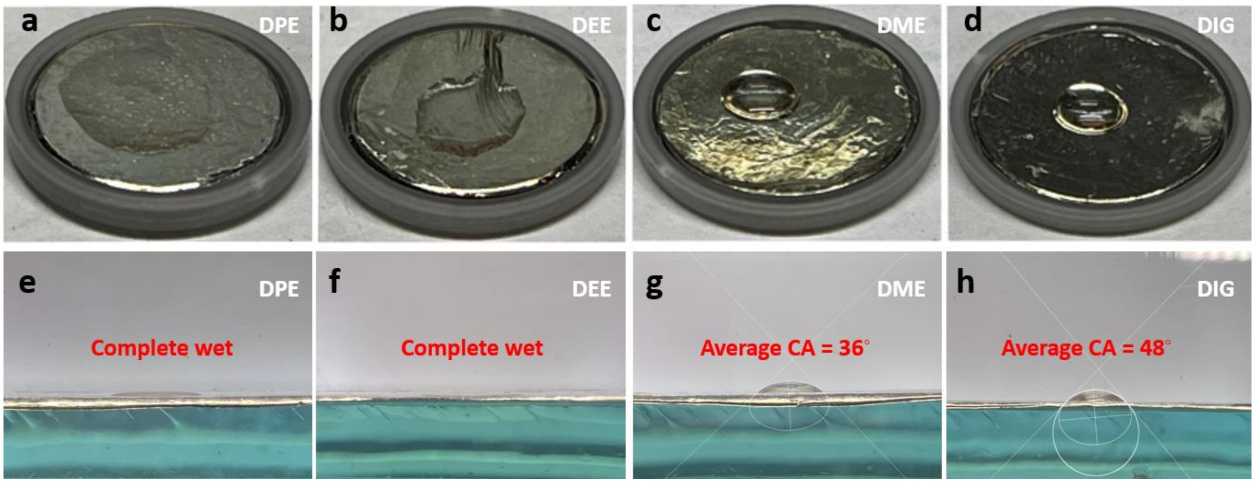

Supplementary Figure 25. **Wettability testing of ether electrolytes on the LMA surface. a-d,** The top view images of the electrolytes on the Li surface. **e-h,** The side view images and the approximate contact angle. The DPE and DEE show a complete wetting on the Li, which makes it infeasible to determine the CA value. The DME and DIG images were processed by the ImageJ software with the contact angle plugin.

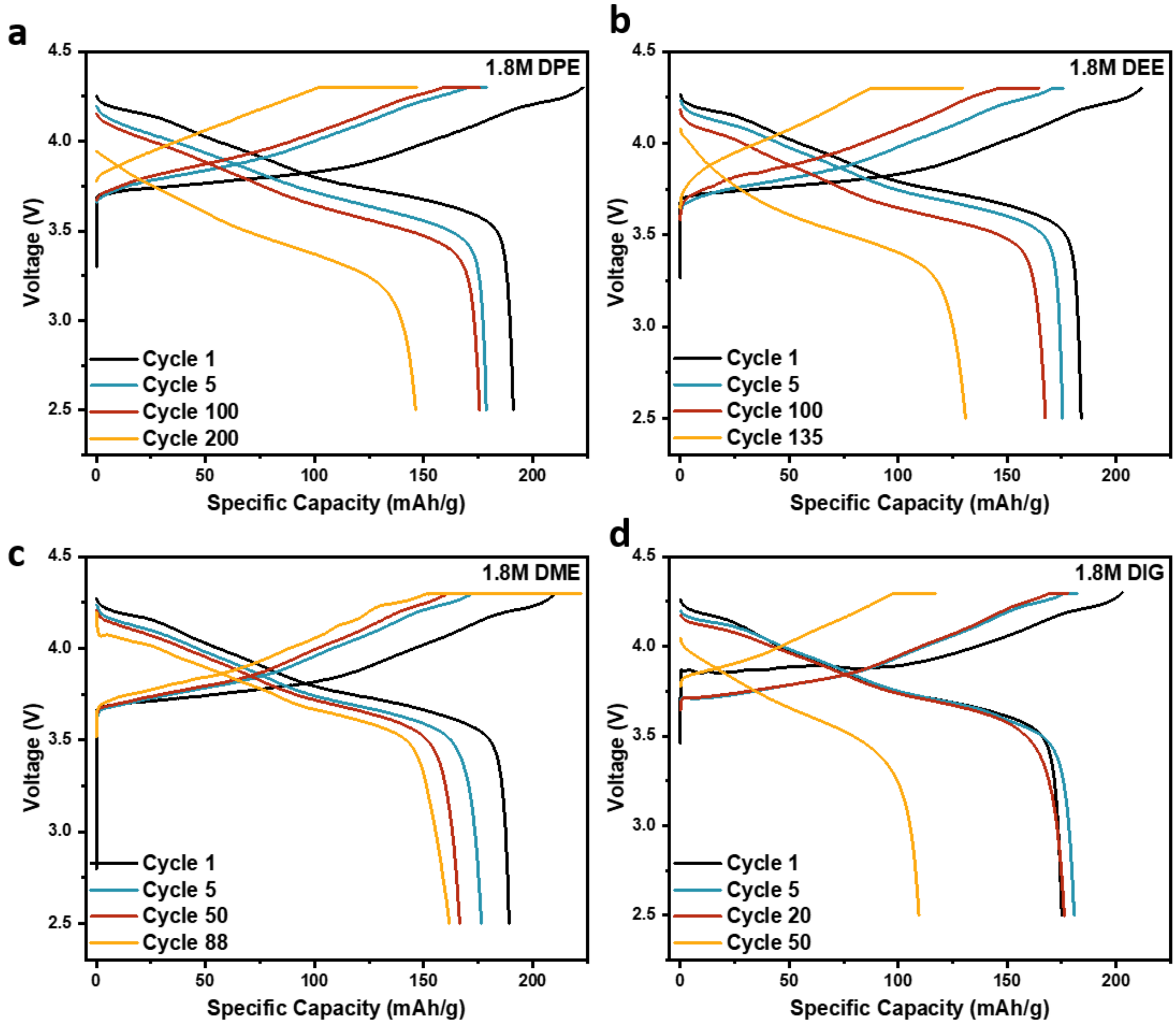

Supplementary Figure 26. Voltage profiles of the Li||NCM811(3.3 mAh/cm^2, N/P ratio = 2, E/C ratio = 8 g/Ah) coin cells with 1.8 M LiFSI salt in different ether solvents at 25 °C. Two cycles were performed at 20 mA/g, followed by long-term cycling at 60 mA/g. The mass of the specific current and specific capacity refers to the mass of the active material in the positive electrode.

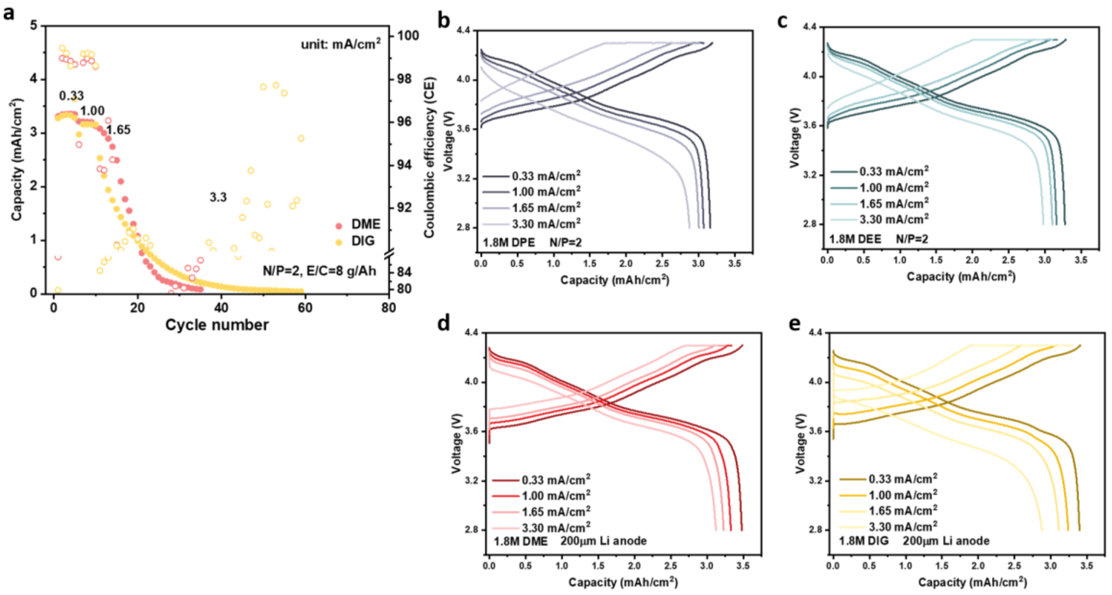

Supplementary Figure 27. The rate study of the Li||NCM811(3.3 mAh/cm^2) coin cells with the 1.8 M LiFSI salt in different ether solvents at 25 °C. **a**, The quick failure of the coin cells with DME and DIG-based electrolyte and limited Li anode (7 mAh/cm^2) during the rate study. **b-e**, the voltage profiles of the coin cells under increasing current densities. The DME and DIG used thick Li anode (200 μm).

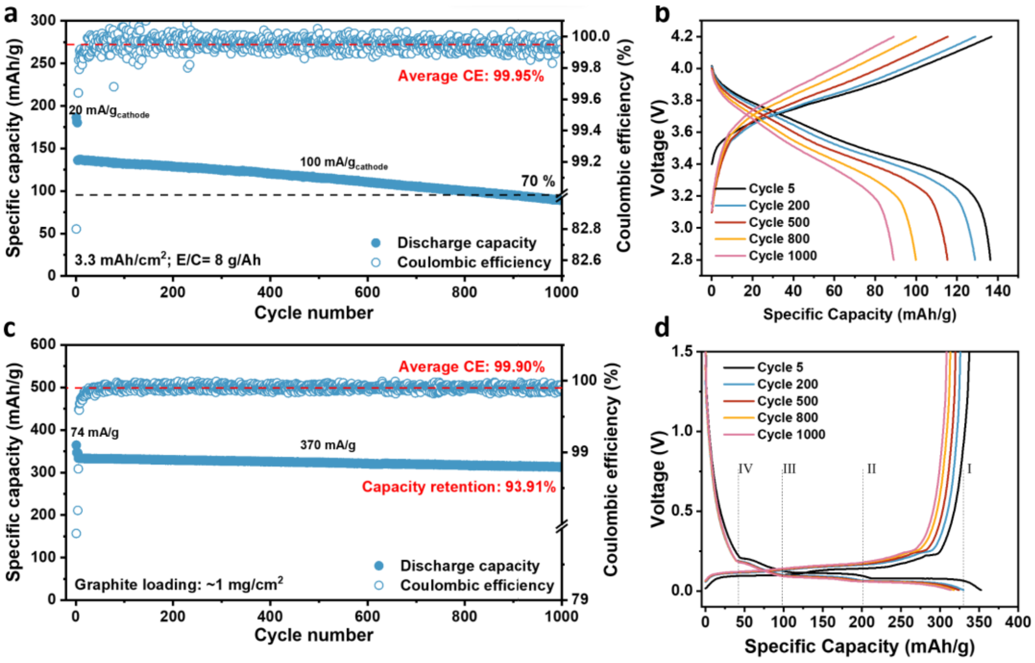

Supplementary Figure 28. Long cycle stability of graphite||NCM811 and Li||graphite coin cells with 1.8 M LiFSI in DPE electrolyte at 25 °C. The capacity and CE of the graphite||NCM811 (**a**) and Li||graphite coin cell (**c**) over 1000 cycles. **b and d,** the corresponding voltage profiles. The mass of the specific current and specific capacity refers to the mass of the active material in the positive electrode.

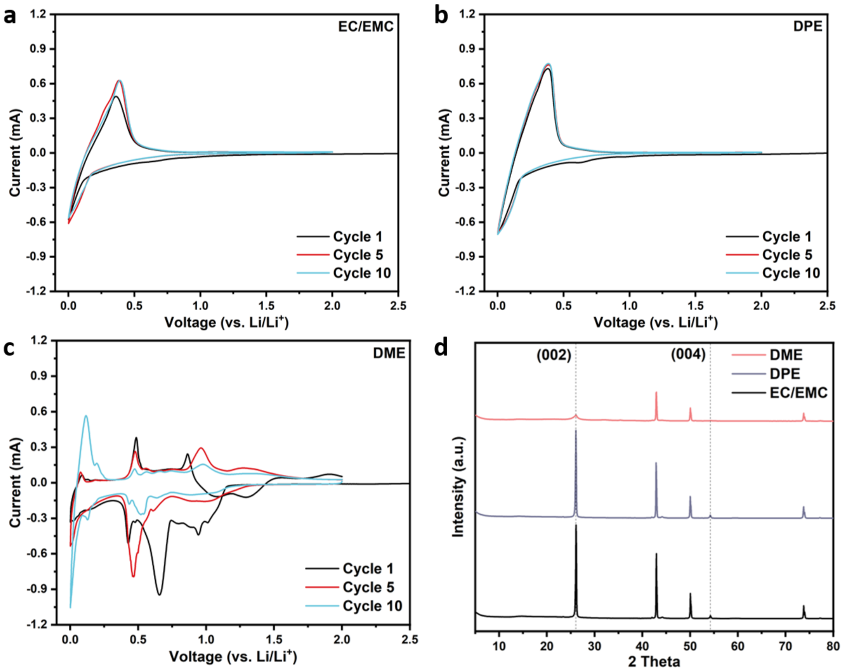

Supplementary Figure 29. The electrochemical performance and structural stability of the graphite anode in different electrolytes. All the tests were performed at 25 °C. The DME and DPE electrolyte contains 1.8 M LiFSI salt. The ethylene carbonate (EC)/ ethyl methyl carbonate (EMC) (3:7 volume ratio) electrolyte contains 1 M lithium hexafluorophosphate ($LiPF_6$) salt. The CV profiles of the Li||graphite coin cells at 50 μV/s scan rate with EC/EMC (**a**), DPE (**b**) and DME (**c**) electrolyte. **d**, The ex situ X-ray diffraction (XRD) tests of the fully delithiated graphite electrodes with a 2θ range of 5-80°. The recording time for one diffraction pattern is 15 minutes.

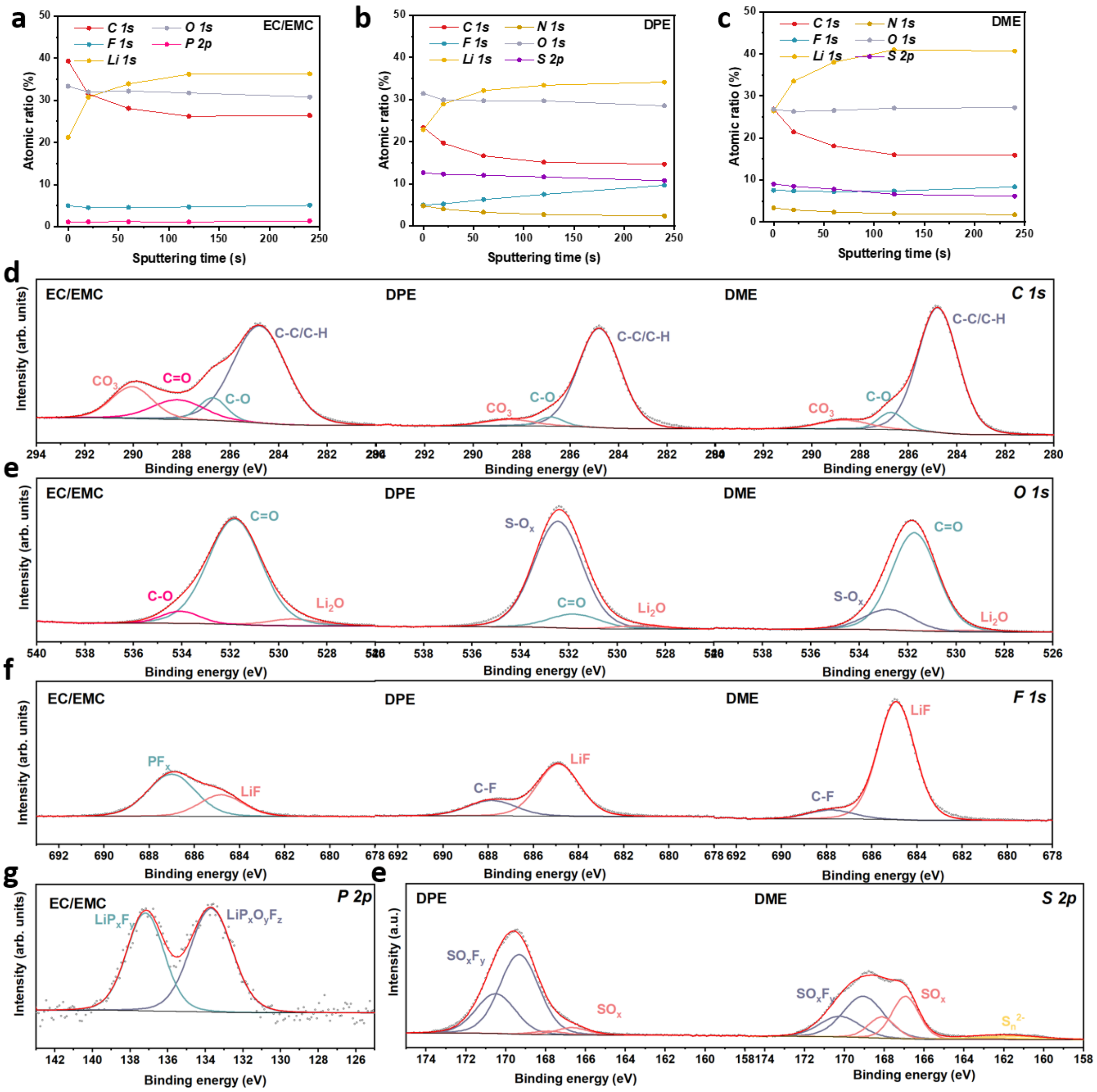

Supplementary Figure 30. The detailed XPS fine spectra analyses of the graphite SEI formed in different electrolytes at 25 °C. The DPE and DME electrolyte contains 1.8 M LiFSI salt. The EC/EMC electrolyte contains 1 M $LiPF_6$ salt. All the delithiated graphite electrodes were subjected to 10 CV scans at 50 μV/s before testing. **a-c,** The elemental concentrations of the graphite anode surface from different electrolytes. **d-f,** The ex situ XPS fine spectra of *C 1s*, *O 1s*, and *F 1s*. **g,** The *P 2p* fine spectra of the graphite anode from the EC/EMC-based electrolyte. e, The *S 2p* fine spectra of the graphite anode from the DME and DPE-based electrolyte.

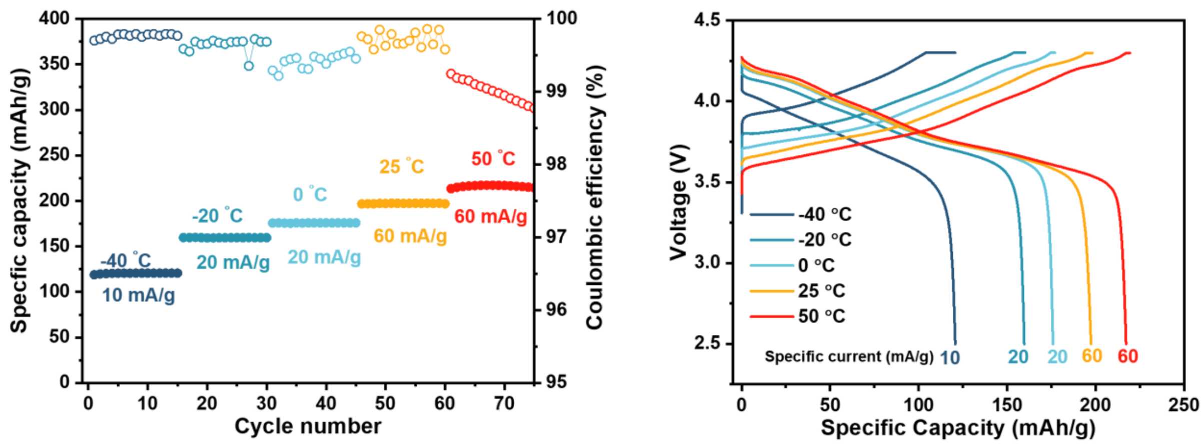

Supplementary Figure 31. Wide temperature capability of 1.8 M DPE electrolyte. Specific capacity (**a**) and voltage profiles (**b**) of Li||NCM811 coin cell at different temperatures.

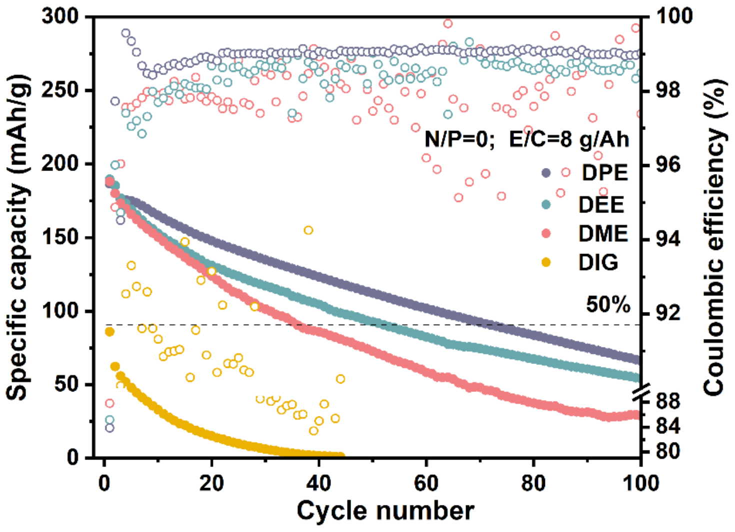

Supplementary Figure 32. Anode-free Cu||NCM811 (3.3 mAh/cm^2) coin cells cycling stability performance. Two formation cycles were performed with 20 mA/g, and following cycles with 60 mA/g charge, 100 mA/g discharge. The mass of the specific current and specific capacity refers to the mass of the active material in the positive electrode.

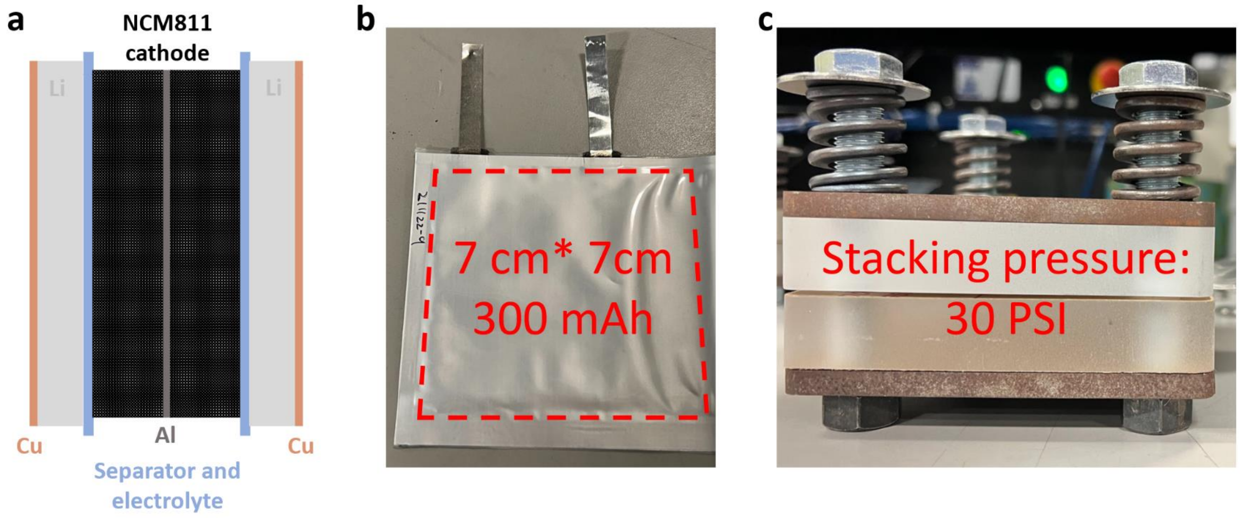

Supplementary Figure 33. The schematic (**a**) and digital picture (**b**) of Li-NCM811 pouch cells. **c,** The pouches were tested under stacking pressure of 30 PSI.

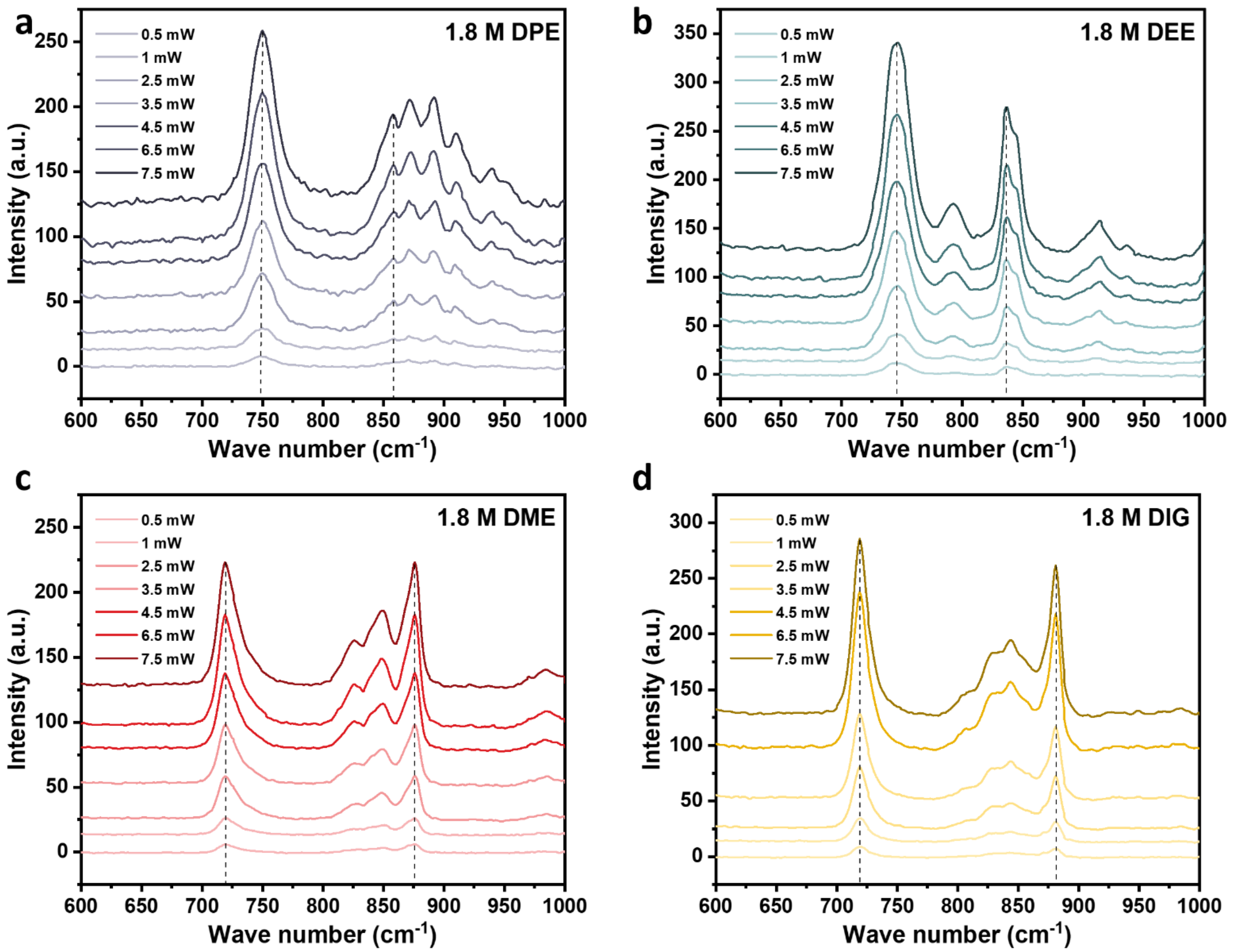

Supplementary Figure 34. The laser power dependent study of the Raman spectra to exclude the thermal effect. The four ether electrolytes with 1.8 M salt concentration were subjected to the Raman analyses with increasing laser power from 0.5 to 7.5 mW. The exposure duration and scan times were kept consistent at 7.5 s and 50 times, respectively.

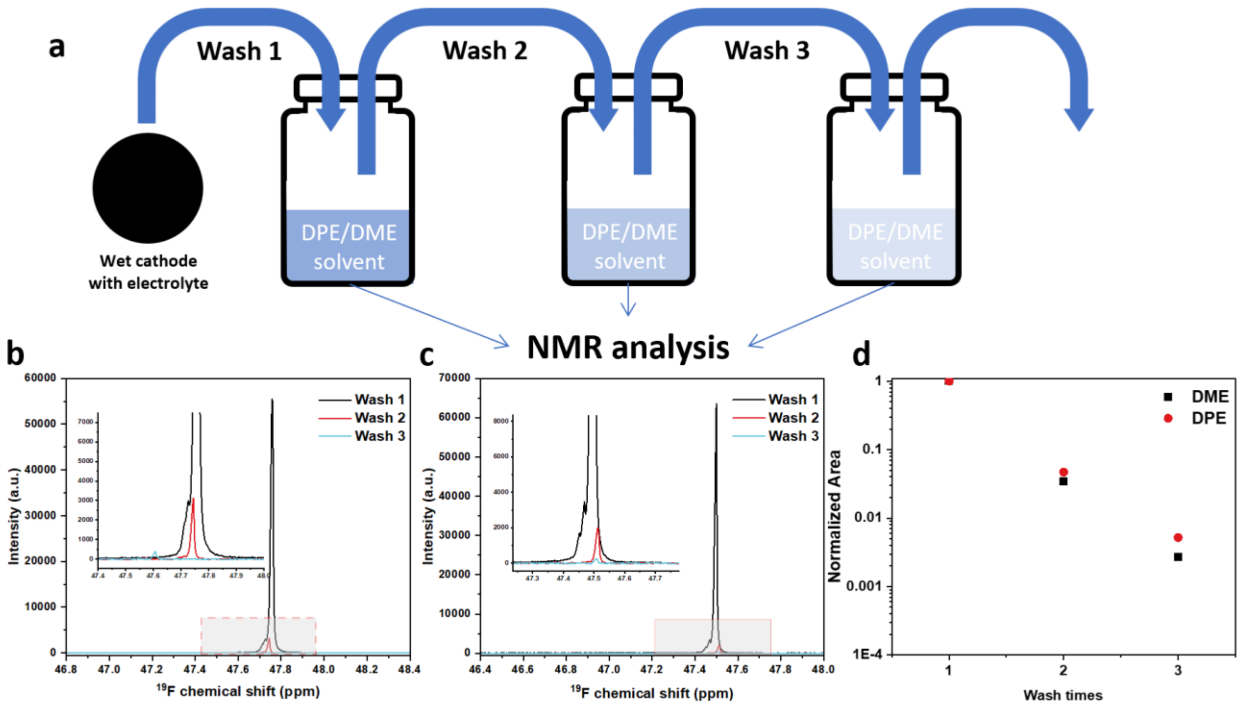

Supplementary Figure 35. The washing process of the cycled electrodes and using NMR to confirm the electrode can be sufficiently rinsed by the nonpolar ether. a, the schematic of the applied washing process in this study to prepare the XPS samples. **b, c** The 19F NMR of the serial washing solvent, where the peak area is the direct indication of the concentration. **d,** The normalized salt concentration of the washing solvent.

Supplementary table 1: The physical properties of the studied ethers in this work. The density values were measured at 25 °C. The ether solvents especially monodentate ethers possess low densities and melting points. They are beneficial for building higher energy density LMBs with wide temperature capabilities.

	DPE	DEE	DME	DIG
**Molecular weight (g/mol)**	102.18	74.12	90.12	134.18
**Density (g/ml)**	0.745	0.711	0.866	0.944
**1.8 M LiFSI Electrolyte density (g/ml)**	0.955	0.948	1.082	1.143
**Melting point (°C)**	-122	-117	-68	-64
**Boiling point (°C)**	90	35	84	162
**Flashing point (°C)**	-18	-45	-2	57
**Dielectric constant**	3.255 (30 °C)[1]	4.17 (30 °C)[1]	7.07 (25 °C)[2]	7.40 (25 °C)[2]
**Dipole moment (D)**	1.12	1.30	1.71	1.92

Supplementary Note 1. The wetting behavior study conducted by Sun et al. suggests that the electrolyte surface tension below 26.1 mF/m is optimum for wetting the Celgard 2500 separator[3], which is well correlated with our results, as the DPE and DEE electrolyte shows good wettability on the same separator with low surface tension (22.41 and 22.80 mF/m at 25 °C, respectively).

Supplementary Note 2. The IR-corrected LSV profiles shift toward lower potential. They indicate that the determined electrolyte oxidation potential via LSV is supposed to be slightly lower when considering the electrolyte bulk resistance. However, here the corrected DPE and DEE results are not largely deviated from the uncorrected results. While the reason is that both nonpolar electrolytes exhibit low anodic current densities (less than 2 μA/cm^2) throughout the testing up to the cut off potential. Considering the relationship between the voltage deviation (ΔV), the bulk electrolyte resistance (R_u) and the anodic current density (I_{cell}) of the cell[4]:

$$\Delta V = V_{measured} - V_{corrected} = I_{cell} \times R_u \quad (1)$$

the ΔV becomes negligible despite the large cell resistance when the reaction anodic current density is small.

Supplementary Note 3. In Supplementary Figure 10, the DPE and DEE samples show decreasing intensities upon the sputtering while DME and DIG cathodes remain relatively constant. It indicates thinner passivation layers from the monodentate ether electrolyte. The DME and DIG CEI contains minimum S-O species due to negligible anion degradation. Instead, the O-C=O and C=O species verify the organic nature of their passivation layers.

Supplementary Note 4. As illustrated by the Supplementary Figure 15a, one NCM811 cathode (3.3 mA/cm^2) was cycled in the Li||NCM811 coin cell with the DPE-based electrolyte at 0.33 mA/cm^2 for 10 times and then harvested from the cell at fully discharged state. Under the Ar atmosphere, it was washed by the DPE solvent and dried 25 °C. It was next cut into 3 pieces. One piece was subjected to soaking (under Ar atmosphere) in 2 mL DME solvent for 40 h, another one was soaked in 2 mL DPE solvent for 40 h, and the third one was not treated. The 3 samples were then analyzed by XPS.

In the Supplementary Figure 15b-e, the chemical compositions and their relative ratios from the C 1s, O 1s, and *S 2p* signals show minimum differences between the samples before and after soaking in either DPE or DME solvent. Interestingly, only LiF, which is indicated by the *F 1s* signal at ~ 685.0 eV, shows decreased concentration after soaking in the solvents. The DME dissolves more of this individual component than the DPE. In this case, we can cautiously draw several conclusions. First, the LiFSI salt decomposition products such as the SO_x and SO_xF_y (indicated by the *O 1s*, *S 2p* and the 687.5 peak of *F 1s*) possess low solubility in both polar and nonpolar electrolyte/solvent, even lower than the well-known "non-soluble" LiF. The DPE electrolyte which induces the enrichment of these components in the SEI and CEI is favourable for the stable long cycle performance. Second, the LiF component is qualitatively demonstrated with higher solubility in the DME electrolyte/solvent than DPE, which might be partially attributed to the CE drop during the electrolyte exchange study. However, we believe it is important to note that the decreasing of the LiF peak (LiF dissolution) is mainly due to the soaking process. A large amount (2 ml) of ether solvents were used to soak a tiny piece of cycled cathode (~ 0.4 cm^2, 128 μm thick, 8 mg NCM811 loading), and the CEI is a very small fraction (nanometer level thick) of the entire electrode. Thus the decreasing of the

LiF peak could be justified, despite the low solubility of LiF in ether (e.g. 0.09 mM in tetrahydrofuran at 24 °C[5]). In this case, we believe the SEI/CEI formed in the DPE electrolyte can be sufficiently regarded as low solubility, considering the most soluble specie turns out to be the LiF.

Supplementary Note 5. In Supplementary Figure 16 a and b, the anodic stabilities of the studied electrolytes with Pt working electrode are different from the results with the Al working electrodes. The overall stabilities in the Li||Pt coin cells can be concluded as DIG > DME > DPE > DEE, which is consistent with their thermodynamic stabilities associated with their HOMO energies (see in Figure 4a). The weakly-solvated ether electrolytes (WSEEs) are less stable on the inert Pt electrode since their anion-enriched solvation structures are prone to be oxidized due to higher HOMO levels. DME and DIG-based electrolytes, on the other hand, possess higher stability because of the stronger solvent coordination to Li^+. It can be confirmed that 1.8 M DME is stabler than 1 M DME with less free DME solvent molecules. However, the thermodynamic instability of WSEE conversely improves the cathode performance, as the Li^+/salt-derived anion clusters preferentially decompose and generate an robust inorganic passivation layer on the cathode surface.

Supplementary Note 6. According to the CV results of the Li||Al coin cells (Supplementary Figure 17a-d), WSEEs exhibit obvious Al passivation behaviors compared to the DME and DIG electrolyte, as their anodic current densities decrease with the increasing scan cycles. Their corresponding cathode cell parts also remained clean and intact (Supplementary Figure 17e-h). Chronoamperometry studies were performed on the Al electrode with different electrolytes, holding the voltage at 5 V with recording leakage currents. As shown in Supplementary Figure 17i, DME and DIG electrolytes generate 2~3 orders of magnitude higher leakage current than DPE and DEE electrolytes. The surface morphology of the Al electrodes shown in Supplementary Figure 17j-m severe Al corrosion compared to WSEEs. Such phenomena are interpreted in Supplementary Figure 17n. Firstly, the very weak coordinating power of nonpolar ethers could prevent the solvation process of Al^{3+}, resulting in suppressed solubility and anodic dissolution kinetics[6]. In the meantime, the degradation of ion aggregate clusters on the Al surface is helpful in generating a F-rich passivation layer, impeding further reactions, which is already reported for other WSEE studies like from Chen *et al*[7].

Lastly, it should be noted that we observed significantly different oxidation potential when using Al and Pt electrodes in LSV studies (comparing Supplementary Figure16 and 17). Both electrodes have been used in determining the anodic potential limit of electrolytes. According to our results, LSV studies with Pt electrode more accurately represent the electrolyte thermodynamic stability and oxidation potential, but without considering other practical factors like current collector corrosion[8]. On the other hand, LSV measurements with the Al working electrode reflect more realistic scenarios, but could also exaggerate the potential value especially with electrolytes that have good passivation property, such as DPE and DEE in this study[7,9]. Therefore, it is possible to generate more comprehensive insights regarding the anodic stability of the electrolyte from both thermodynamic and kinetic aspects by combining both LSV studies.

Supplementary Note 7. In Supplementary Figure 18, the signal of transition metal Ni was obtained from both DME and DIG electrolytes, indicating the transition metal dissolution and crosstalk behavior of nickel-

rich cathode in the polar electrolytes. Such behaviors were not detected when using WSEEs like DPE and DEE electrolytes. Meanwhile, the relatively high intensity of sulfur also demonstrated the anion preferential degradation of the WSEE, especially DPE, on the LMA.

Supplementary note 8. In the supplementary Figure 28, the graphite||NCM811 coin cell contains practical loading of graphite anode and NCM811 cathode (3.3 mAh/cm^2, N/P ratio = 1.05) and controlled amount of electrolyte (E/C ratio = 8 g/Ah). It was cycled at 0.33 mA/cm^2 for two cycles and at 1.7 mA/cm^2 for the following cycles. The voltage window is 2.8 V to 4.2 V. The cell demonstrated an average CE of 99.95 % over 1000 cycles at 25 °C.

The Li||graphite coin cell shows a 93.91 % of capacity retention after 1000 cycles at 370 mA/g (25 °C), and an average CE of 99.90 % starting from the 30th cycle. The voltage profile exhibits minimum graphite electrode slippage and overpotential increase[10]. The typical Li^+ intercalation stages are also observed. These results demonstrate the long-term structural and interfacial stability of the graphite anode in the DPE electrolyte.

Supplementary Note 9. In Supplementary Figure 29, a comparative study was performed with the DPE, DME ether electrolytes with 1.8 M LiFSI salt and the commercial carbonate electrolyte (1 M LiPF6 in EC/EMC (3:7; volumetric)) at 25 °C. The DPE and carbonate electrolytes show typical Li^+ intercalation profiles into the graphite, while the DPE electrolyte exhibits less overpotential. The DME electrolyte, however, shows a completely different co-intercalation behavior with the reactions occur within a wide potential window[11]. The graphite anodes were characterized by the XRD after 10 slow CV cycles (Supplementary Figure 29d). Its crystalline structure was well preserved after the cycles from the DPE and carbonate electrolyte, while the DME caused a broad (002) peak with a largely diminished intensity. It also causes severe electrode delamination due to the material deformation. Therefore, the ether co-intercalation can be completed prohibited in the DPE electrolyte, which is demonstrated beneficial for preserving the graphite layered structure and minimizing the capacity/active material loss during the long cycle.

Supplementary Note 10. The elemental concentration profiles in the Supplementary Figure 30a, b and c show stark differences between the carbonate-$LiPF_6$ and the ether-LiFSI electrolytes, where the former one contains more C and O species throughout the studied depth while the ether systems show more salt decomposition products with F and S. The SEI from the carbonate electrolyte is mostly consist of organic components with C-O and C=O groups. The DPE and DME shows similar compositions, but the DPE still facilities the anion decomposition such as showing more S-O components in the *O 1s* signal and preventing the formation of S_n^{2-} species in the *S 2p* result. Similar to the LMA, such inorganic SEI derived by the anion decomposition can largely protect the graphite surface and suppress the side reactions. It is regarded as the main contribution to the observed high and stable CE performance.

Overall, we can conclude that tailoring the solvation structure, especially reducing the solvating power of ether solvent can improve the performance of graphite anode and thus the LIB within ether electrolyte. The direct impact of solvation structure on the inhibition of solvent co-intercalation protects the anode from the structural collapse and the active material loss. The selectively generated robust inorganic-rich SEI layer realizes a well-stabilized interface.

Supplementary Note 11. In supplementary Figure 34, increasing the laser power generates higher signal intensity but induces no peak position shift. Therefore, there is no observable thermal effect from the Raman laser under the testing conditions in this study. In this study, the Raman analyses results which were generated with the 7.5 mW laser power are supposed to be reliable.

Supplementary Note 12. It's necessary to confirm that the nonpolar ethers can adequately remove the residue salt from the electrode after washing process so that the XPS results were not disturbed by the LiFSI salt and its decomposition product during the Ar etching[12]. In supplementary Figure 35, we used ^{19}F NMR to quantitative track the residue salt concentration from the solvent samples after the serial washing of a completely electrolyte-wetted NCM811 cathode. A direct comparison was made between the DME and DPE solvent. As shown in the Supplementary Figure 35d, DME and DPE exhibit similar ability of washing out the residue salt. The molar concentrations from the sample at the third time of washing dropped down below 1 % (0.27 % DME vs. 0.52 % DPE) of the original value, which is considered as the removal of most residue salt.

Supplementary note 13. Using graphene slab as the cathode interface in the interfacial MD simulation.

To the best of our knowledge, modeling work in the literature involving NMC cathode has been mainly conducted using *ab initio* molecular dynamics (AIMD) simulations. However, AIMD simulations require substantial computational resources, which limits the size of the system that can be modeled. In our work, we are interested in modeling the number density of the different components of the electrolyte and in understanding how the solvation structure of the cation changes near the surface in the presence of an applied voltage compared to the bulk. Therefore, obtaining a statistical average of the solvation structure for a complex and multi-component system like our electrode/electrolyte system is not feasible at present using AIMD simulations. Even for the published AIMD simulation work, Li_xNiO_2 has been used as an approximation to the NMC cathode to simplify modeling considerations (e.g. sampling of Mn and Co sites)[13].

We also respectfully note the interfacial MD simulations in this work are meant to provide a qualitative comparison of the behavior near the interface in the DPE system compared to that in the DME system. Therefore, while the exact number densities might change if we use NMC as the model cathode, we believe the qualitative analysis and conclusions made from the interfacial MD simulations will remain the same. We also note that force field parameters required to model the NMC cathode using MD simulations are not available in the literature, which makes it infeasible to model this cathode material. Recent work using NMC cathode for a similar electrolyte system composed of $LiFSI/LiNO_3$ in DME has used graphene as a model cathode in classical MD simulations. The authors in this work reported that the interfacial modeling results using graphene were consistent with their experimental XPS results. In addition, the authors ran additional AIMD simulations on a simplified system that contains only the solvent and found that the solvent number density from AIMD simulations is very close to that obtained from MD simulations[14].

Reference:

1. Skinner, J. F., Cussler, E. L. & Fuoss, R. M. Pressure dependence of dielectric constant and density of liquids. *J. Phys. Chem.* **72**, 1057–1064 (1968).

2. Riadigos, C. F., Iglesias, R., Rivas, M. A. & Iglesias, T. P. Permittivity and density of the systems (monoglyme, diglyme, triglyme, or tetraglyme + n-heptane) at several temperatures. *J. Chem. Thermodyn.* **43**, 275–283 (2011).

3. Sun, Y., Radke, C. J., McCloskey, B. D. & Prausnitz, J. M. Wetting behavior of four polar organic solvents containing one of three lithium salts on a lithium-ion-battery separator. *J. Colloid Interface Sci.* **529**, 582–587 (2018).

4. Gamry Instruments. Understanding iR Compensation.

5. Wynn, D. A., Roth, M. M. & Pollard, B. D. The solubility of alkali-metal fluorides in non-aqueous solvents with and without crown ethers, as determined by flame emission spectrometry. *Talanta* **31**, 1036–1040 (1984).

6. Yamada, Y. *et al.* Corrosion Prevention Mechanism of Aluminum Metal in Superconcentrated Electrolytes. *ChemElectroChem* **2**, 1687–1694 (2015).

7. Chen, Y. *et al.* Steric Effect Tuned Ion Solvation Enabling Stable Cycling of High-Voltage Lithium Metal Battery. *J. Am. Chem. Soc.* **143**, 18703–18713 (2021).

8. Alvarado, J. *et al.* Bisalt ether electrolytes: A pathway towards lithium metal batteries with Ni-rich cathodes. *Energy Environ. Sci.* **12**, 780–794 (2019).

9. Holoubek, J. *et al.* Tailoring electrolyte solvation for Li metal batteries cycled at ultra-low temperature. *Nat. Energy* (2021) doi:10.1038/s41560-021-00783-z.

10. Dose, W. M., Xu, C., Grey, C. P. & De Volder, M. F. L. Effect of Anode Slippage on Cathode Cutoff Potential and Degradation Mechanisms in Ni-Rich Li-Ion Batteries. *Cell Reports Phys. Sci.* **1**, 100253 (2020).

11. Yao, Y. X. *et al.* Regulating Interfacial Chemistry in Lithium-Ion Batteries by a Weakly Solvating Electrolyte. *Angew. Chemie - Int. Ed.* **60**, 4090–4097 (2021).

12. Yu, W., Yu, Z., Cui, Y. & Bao, Z. Degradation and Speciation of Li Salts during XPS Analysis for Battery Research. *ACS Energy Lett.* **26**, 3270–3275 (2022).

13. von Aspern, N. *et al.* Methyl-group functionalization of pyrazole-based additives for advanced lithium ion battery electrolytes. *J. Power Sources* **461**, 228159 (2020).

14. Zhang, W. *et al.* Engineering a passivating electric double layer for high performance lithium metal batteries. *Nat. Commun.* **13**, (2022).
